# Activation of adenosine A_2A_ receptor by lipids from docosahexaenoic acid revealed by NMR

**DOI:** 10.1126/sciadv.aay8544

**Published:** 2020-03-18

**Authors:** Takuya Mizumura, Keita Kondo, Masatoshi Kurita, Yutaka Kofuku, Mei Natsume, Shunsuke Imai, Yutaro Shiraishi, Takumi Ueda, Ichio Shimada

**Affiliations:** 1 Graduate School of Pharmaceutical Sciences, The University of Tokyo, Tokyo, Japan.; 2 Precursory Research for Embryonic Science and Technology, Japan Science and Technology Agency, Kawaguchi, Japan.

## Abstract

The lipid composition of the plasma membrane is a key parameter in controlling signal transduction through G protein–coupled receptors (GPCRs). Adenosine A_2A_ receptor (A_2A_AR) is located in the lipid bilayers of cells, containing acyl chains derived from docosahexaenoic acid (DHA). For the NMR studies, we prepared A_2A_AR in lipid bilayers of nanodiscs, containing DHA chains and other acyl chains. The DHA chains in nanodiscs enhanced the activation of G proteins by A_2A_AR. Our NMR studies revealed that the DHA chains redistribute the multiple conformations of A_2A_AR toward those preferable for G protein binding. In these conformations, the rotational angle of transmembrane helix 6 is similar to that in the A_2A_AR–G protein complex, suggesting that the population shift of the equilibrium causes the enhanced activation of G protein by A_2A_AR. These findings provide insights into the control of neurotransmissions by A_2A_AR and the effects of lipids on various GPCR functions.

## Teaser

NMR studies of A_2A_AR in lipid bilayers of nanodiscs revealed the mechanism underlying activation of A_2A_AR by DHA chains.

## INTRODUCTION

G protein–coupled receptors (GPCRs) are one of the largest membrane protein families in eukaryotes. The GPCR family includes receptors of various neurotransmitters and hormones, and more than 30% of modern drugs target GPCRs. Drug binding to GPCRs leads to the induction or inhibition of signal transduction mediated by G proteins, β-arrestins, and various other effectors. Under physiological conditions, GPCRs are embedded in lipid bilayers, and accumulating evidence has shown that the signaling activities of GPCRs are affected by the surrounding lipids.

Adenosine A_2A_ receptor (A_2A_AR), a class A GPCR, controls inflammation, neurotransmission, blood flow, and immune responses (*1*). In the brain striatum, A_2A_AR is involved in neurotransmissions that controls motor skills, and A_2A_AR is a drug target for the reduction of dyskinesia in Parkinson’s disease (*1*).

In cells, A_2A_AR is embedded in membranes containing acyl chains derived from docosahexaenoic acid (DHA), a polyunsaturated fatty acid with 22 carbons and six double bonds, and arachidonic acid (ARA), a polyunsaturated fatty acid with 20 carbons and four double bonds. Dietary DHA intake increases and decreases the populations of the DHA and ARA chains, respectively (*2*) . In the lipid bilayers of the mammalian brain striatum, in which A_2A_AR is highly expressed (*1*), DHA constitutes 12 to 14% of the phospholipid acyl chains, and the population of the DHA chains is remarkably decreased from 3 to 8% by feeding DHA-deficient diets (*3*, *4*). The effects of DHA on the activities of A_2A_AR are unknown, although the signaling activities of rhodopsin and cannabinoid CB1 receptor, which share ~10% sequence identity with A_2A_AR, are reportedly affected by the population of the DHA chains in the lipid bilayer (*5*, *6*). Clarification of the effects of DHA on the conformation and activity of A_2A_AR would be helpful for understanding the signal transduction through A_2A_AR under physiological conditions.

The three-dimensional structures of A_2A_AR have been solved in various forms, including the inverse agonist bound, full agonist bound, and ternary complex composed of A_2A_AR, a full agonist, and an engineered G protein, by x-ray crystallography and cryo–electron microscopy (*7*–*10*). In the structures of the ternary complex, the cytoplasmic half of transmembrane 6 (TM6) was shifted outward and rotated clockwise, when viewed from the cytoplasmic surface, to form a cavity in the cytoplasmic surface, as compared to the structures in the inverse agonist-bound form. The C-terminal helix of the engineered G protein was inserted into the cavity (*7*, *10*). The cytoplasmic cavities were also found in the crystal structures of other GPCRs bound to G protein or arrestin (*11*). In the solution state, GPCRs exist in an equilibrium between inactive and active conformations, and the populations and exchange rates between these two conformations are related to their functions, as exemplified by nuclear magnetic resonance (NMR) and other spectroscopic studies (*12*). In the case of A_2A_AR, the NMR signals originating from the isoleucine δ1-methyl groups (*13*) and chemically introduced CF_3_ probes (*14*) indicated that the TM region of A_2A_AR exchanges between multiple conformations in the presence of the full agonist. In addition, the resonances from the glycine NH and tryptophan NεH groups of A_2A_AR were used to observe the allosteric modulation of W246 by the D52N mutation (*15*). However, little is known about the effects of the DHA chains on the conformations and the signaling activities of GPCRs. Therefore, the function-related dynamics of A_2A_AR in lipid bilayers containing DHA chains must be clarified.

Reconstituted high-density lipoproteins (rHDLs), also known as nanodiscs (*16*), can accommodate membrane proteins within a 10-nm-diameter disc-shaped lipid bilayer. rHDLs reportedly provide lipid environments with more native-like properties, as compared with liposomes, in terms of the curvature and phase behavior (*17*). rHDLs and other disc-shaped lipid bilayers have been used in various in vitro GPCR studies (*18*–*21*), and our NMR studies of GPCRs in rHDL lipid bilayers revealed that the populations of the active conformation of GPCRs in rHDLs correlated better with the signaling levels than those in detergent micelles (*20*). These methods for the reconstruction of GPCRs into the lipid bilayers of rHDLs allow examination of the effects of the composition of lipid bilayers on the activities and conformational equilibria of GPCRs.

Here, we observed the NMR signals of the methionine residues of A_2A_AR in the lipid bilayers of rHDLs, containing the DHA and ARA chains, to identify the conformational equilibria related to the signaling activities. We found that the DHA chains in the rHDLs enhanced the G protein activation by A_2A_AR. On the basis of our observation of the conformational equilibria, we discuss the mechanism underlying the modulation of the signaling activity of A_2A_AR by the DHA chains.

## RESULTS

### Preparation and characterization of A_2A_AR in rHDL

**Fig. 1:**
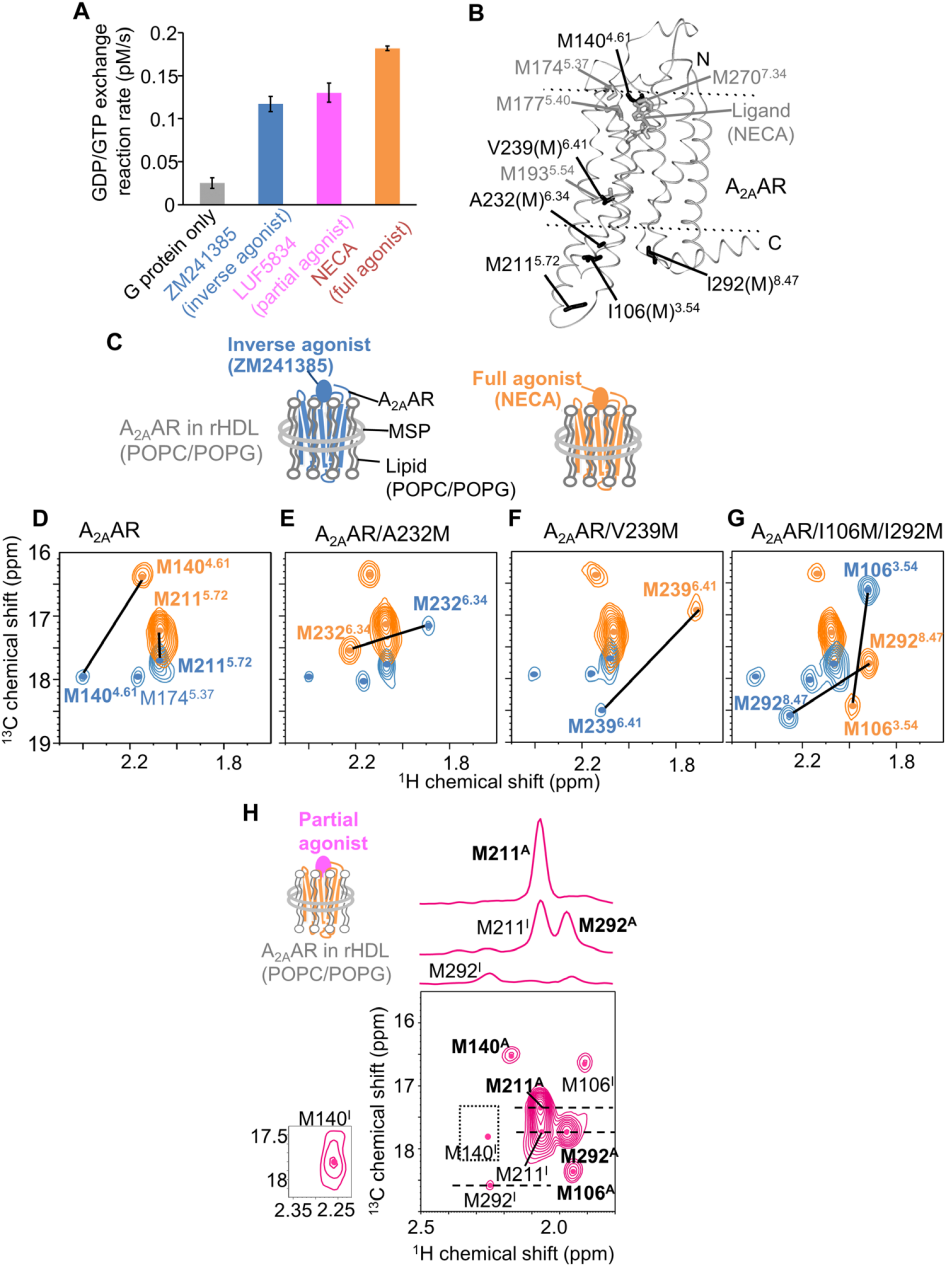
Conformations of A_2A_AR in rHDLs bound to the inverse agonist, full agonist, and partial agonist. (**A**) [^35^S]-GTPγS binding to complexes of the purified G protein, A_2A_AR in rHDL(POPC/POPG), and the ligands. Results are expressed as the initial rates of the production of the GTPγS and G protein complex. Data are the means ± SEM of triplicate determinations from three separate representative experiments. (**B**) Distribution of the residues with NMR signals observed for A_2A_AR in rHDLs in the crystal structure of A_2A_AR. The crystal structure of A_2A_AR with a full agonist, NECA [Protein Data Bank (PDB) code: 2YDV], is shown in ribbons in a side view with the extracellular sides on the top. I106(M)^3.54^, M140^4.61^, M211^5.72^, A232(M)^6.34^, V239(M)^6.41^, and I292(M)^8.47^ are depicted by black sticks. M174^5.37^, M177^5.40^, M193^5.54^, and M270^7.34^ are depicted by gray sticks. NECA is depicted by gray sticks. The membrane position generated by the Orientations of Proteins in Membranes (OPM) database (http://opm.phar.umich.edu/) is indicated. (**C**) Schematic diagrams of A_2A_AR in rHDL(POPC/POPG) bound to the inverse agonist and full agonist. (**D** to **G**) Overlaid ^1^H-^13^C HMQC spectra of A_2A_AR (D), A_2A_AR/A232M (E), A_2A_AR/V239M (F), and A_2A_AR/I106M/I292M (G), labeled with [[α,β,β-^2^H, methyl-^13^C] Met, u-^2^H], bound to the inverse agonist (blue) and the full agonist (orange). Only the regions with methionine methyl resonances are shown. The M140^4.61^ and M211^5.72^ signals in (D), the M232^6.34^ signals in (E), the M239^6.41^ signals in (F), and the M106^3.54^ and M292^8.47^ signals in (G) are indicated and connected with lines. (**H**) ^1^H-^13^C HMQC spectra of [[α,β,β-^2^H, methyl-^13^C] Met, u-^2^H] A_2A_AR/I106M/I292M in rHDL(POPC/POPG) in the presence of the partial agonist. Cross sections at the dashed lines are shown above the spectrum. Superscripts I and A are added to the labels of the M106^3.54^, M140^4.61^, M211^5.72^, and M292^8.47^ signals with chemical shifts similar to those in the presence of the inverse agonist and the full agonist, respectively. The M140^I^ signal, which is lower than that of the minimum contour level, is enclosed in boxes and shown at the left of the spectrum with different contour levels. In (D) to (H), only the regions with methionine methyl resonances are shown, and the centers of the methionine resonances are indicated with dots.

*Pichia pastoris* was used as the host for the large-scale expression of functional A_2A_AR. A_2A_AR was solubilized in *n*-dodecyl-β-d-maltopyranoside (DDM) and purified by two purification steps. For the investigation of the conformational dynamics of A_2A_AR in lipid bilayers, purified A_2A_AR in DDM micelles was reconstituted into rHDLs with a mixture of 1-palmitoyl-2-oleoyl-phosphatidylcholine (POPC) and 1-palmitoyl-2-oleoyl-phosphatidylglycerol (POPG). The population of the anionic lipid (POPG) was 25% (POPC:POPG = 3:1), which is similar to the population of the anionic lipids in mammalian cell membranes (*22*). Here, the rHDLs prepared from the POPC and POPG lipids are referred to as rHDL(POPC/POPG). The size exclusion chromatography revealed that purified A_2A_AR in rHDL(POPC/POPG) is monodisperse, with a Stokes diameter of 12 nm (fig. S1A), in good agreement with the previously reported rHDL size. As judged from SDS–polyacrylamide gel electrophoresis analyses, the purity of A_2A_AR in rHDL(POPC/POPG) was >90%, and the ratio of A_2A_AR to MSP1E3 was consistent with the 1:2 stoichiometry that would be expected when each rHDL particle is composed of two MSP1E3 molecules and one A_2A_AR (fig. S1B). Radioligand-binding assays with an excess amount of a tritium-labeled full agonist, 5′-(*N*-ethylcarboxamido)adenosine (NECA), which has a higher affinity for A_2A_AR than the endogenous ligand adenosine, revealed that more than 80% of the purified A_2A_AR in rHDL(POPC/POPG) retained the NECA binding activity. The ligand-binding activity remaining after a 24-hour incubation at 298 K was ~80% of that of freshly prepared A_2A_AR in rHDL(POPC/POPG) (fig. S1C), suggesting that A_2A_AR in rHDLs is stable during NMR measurements. The dose-dependent curve, observed in the tritium-labeled NECA binding assays, yielded the dissociation constant of 180 nM (fig. S1D), which is comparable to the previously reported value, 430 nM (*23*). The guanosine diphosphate/guanosine triphosphate (GDP/GTP) reaction rates of the complex of A_2A_AR in rHDL(POPC/POPG), the heterotrimeric G protein, and the ligands were in good agreement with the ligand efficacies from a previous study, using A_2A_AR in a cell membrane (*24*), suggesting that the A_2A_AR in rHDLs retained the ability to stimulate the G protein in a ligand-dependent manner (Fig. 1A).

### NMR resonances from methionine residues of A_2A_AR in rHDLs

To observe the NMR resonances from A_2A_AR in rHDLs with a molecular mass of ~180 kDa, composed of ~35 kDa A_2A_AR, ~80 kDa lipids, and ~60 kDa MSP1E3, we used ^13^C selective labeling of the methionine methyl groups, which reflect the spatial arrangement of the neighboring aromatic residues (*12*), along with sensitivity enhancement by deuteration. The deuteration and methionine methyl–selective ^13^C labeling in the yeast expression system were accomplished by using medium containing deuterium oxide and [α,β,β-^2^H, methyl-^13^C] methionine. In the ^1^H-^13^C heteronuclear multiple-quantum coherence (HMQC) spectrum of [[α,β,β,-^2^H, methyl-^13^C] Met, u-^2^H] A_2A_AR in DDM micelles bound to the inverse agonist, resonances that apparently originate from six methionine residues were observed (fig. S2A). For the assignment of the methionine resonances, we mutated the methionine residues. For example, M211^5.72^ [superscripts indicate GPCR database (GPCRdb) numbering (*25*)] was assigned by introducing the M211T mutation into A_2A_AR. A resonance was absent in the spectrum of the M211T mutant, revealing that this resonance is from M211^5.72^ (fig. S2B). The resonances from the other methionine residues were assigned in a similar manner (fig. S2, C to G). Assignments for the methionine residues in the presence of the full agonist were accomplished in the same manner as those in the presence of the inverse agonist (fig. S2, H to L). The resonances from A_2A_AR in rHDLs were assigned by comparison with the spectra of A_2A_AR in micelles. In the spectra of [[α,β,β,-^2^H, methyl-^13^C] Met, u-^2^H] A_2A_AR in rHDL(POPC/POPG) bound to the inverse agonist, the resonances with chemical shifts similar to those of M140^4.61^, M174^4.61^, and M211^5.72^ in DDM micelles were observed, revealing that they are from M140^4.61^, M174^5.37^, and M211^5.72^ (fig. S2M). The resonances corresponding to M140^4.61^ and M211^5.72^ of A_2A_AR in rHDL(POPC/POPG) bound to the full agonist were assigned in a similar manner (fig. S2N).

To investigate the conformation of A_2A_AR at the cytoplasmic surface, we newly introduced methionine residues as structural probes to the cytoplasmic surface by the I106M, A232M, V239M, and I292M mutations (Fig. 1B), according to the comparison with other class A GPCRs: The residues corresponding to I106^3.54^, A232^6.34^, V239^6.41^, and I292^8.47^ are methionine in at least one of the class A GPCRs. We confirmed that these mutants retained the ligand binding activity (fig. S3, A to E) and the G protein signaling activity (fig. S3F) and that these mutants exhibited the ligand binding activities after a 24-hour incubation at 298 K (fig. S3G). In the ^1^H-^13^C HMQC spectra of [[α,β,β,-^2^H, methyl-^13^C] Met, u-^2^H] A_2A_AR in rHDL(POPC/POPG) with the A232M, V239M, I292M, or I106M/I292M mutations, bound to the inverse agonist (fig. S4, A to D) and the full agonist (fig. S4, E to H), one resonance was additionally observed upon the methionine residue introduction, revealing that these resonances are from M232^6.34^, M239^6.41^, M292^8.47^, and M106^3.54^. The remaining methionine methyl signals did not exhibit substantial chemical shift changes in the presence of these mutations, and thus, they did not affect the global conformation of A_2A_AR. The chemical shifts of the resonances from M106^3.54^, M140^4.61^, M211^5.72^, M232^6.34^, M239^6.41^, and M292^8.47^ of A_2A_AR in rHDL(POPC/POPG) in the presence of the inverse agonist were markedly different from those in the presence of the full agonist (Fig. 1, C to G), suggesting that a large conformational change occurs upon A_2A_AR activation.

### Conformational equilibrium of A_2A_AR in rHDLs bound to the full agonist and partial agonist

To examine the resonances undergoing conformational exchanges, we also recorded the spectra at lower and higher temperatures, 288 and 308 K, respectively. In the spectra observed at 288 and 308 K, the resonance from M106^3.54^ remarkably shifted from that at 298 K (fig. S5, A and B). In addition to the resonance from M106^3.54^, the resonances from M140^4.61^ and M292^8.47^ exhibited the temperature-dependent shift (fig. S5, C to F). In the cases of M140^4.61^ and M292^8.47^, the chemical shift changes corresponding to these temperature-dependent shifts were not induced by the type of surrounding lipids (vide infra). Thus, these temperature-dependent shifts reflect other conformational equilibria, which are not regulated by the lipids. The M211^5.72^ signals exhibited almost identical chemical shifts at 288, 298, and 308 K. These results suggest that A_2A_AR in the presence of the full agonist assumes multiple active substates, exchanging faster than the chemical shift difference (>100 s^−1^).

In the ^1^H-^13^C HMQC spectrum of [[α,β,β,-^2^H, methyl-^13^C] Met, u-^2^H] A_2A_AR/I292M in rHDL(POPC/POPG) bound to the partial agonist, LUF5834, M106^3.54^, M140^4.61^, M211^5.72^, and M292^8.47^ exhibited two resonances, and their chemical shifts were similar to those observed when bound to the inverse agonist (M106^I^, M140^I^, M211^I^, and M292^I^ in Fig. 1H) and the full agonist (M106^A^, M140^A^, M211^A^, and M292^A^ in Fig. 1H). The M106^A^ signal exhibited the temperature-dependent shift (fig. S5, G to I). In addition, the intensity ratios of the active state signals (M106^A^, M140^A^, M211^A^, and M292^A^), relative to the inactive state signals (M106^I^, M140^I^, M211^I^, and M292^I^), were decreased at the lower temperatures (fig. S5J). These signal intensity changes of A_2A_AR in the presence of the partial agonist suggest that there is an exchange between the inactive and active states that occurred at a slower rate (<100 s^−1^) than the chemical shift differences, and the temperature-dependent shift of the M106^A^ signal suggests that the active state exists in equilibria among multiple substates, with a faster exchange rate (>100 s^−1^) than the chemical shift differences.

### DHA chains enhanced G protein activation by A_2A_AR

**Fig. 2:**
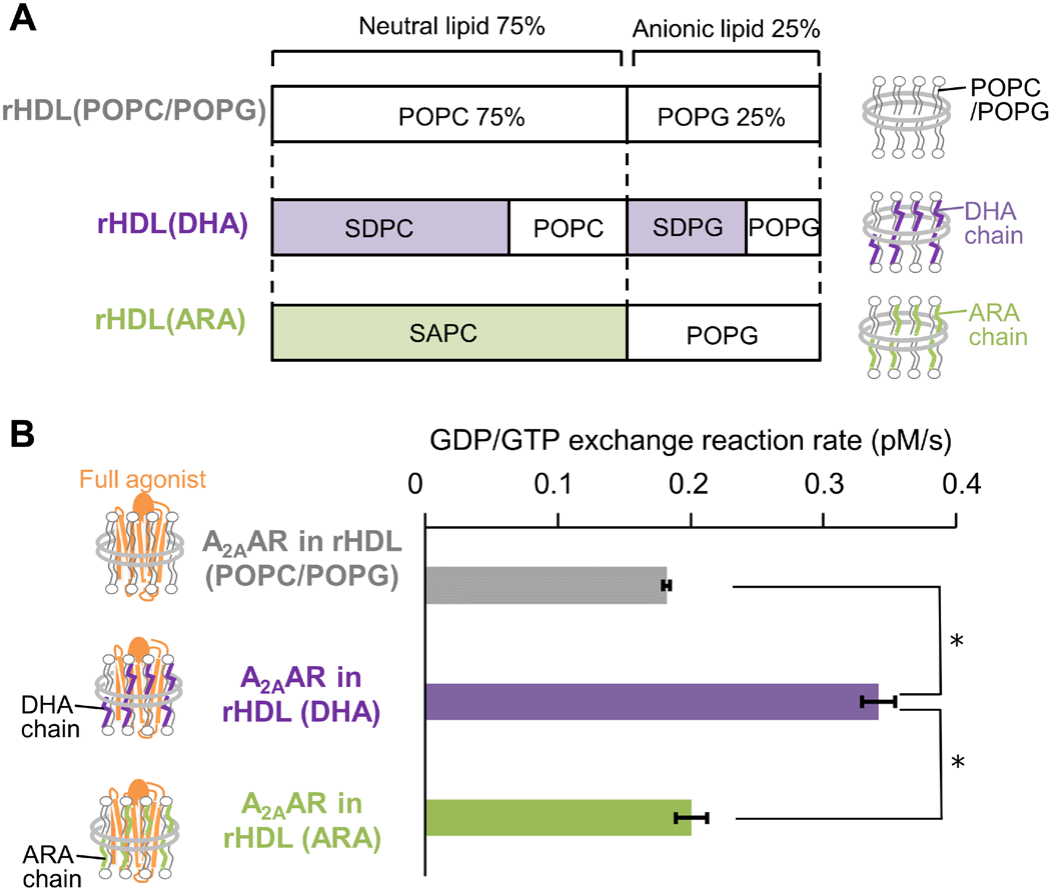
Signaling activities of A_2A_AR in rHDLs with various lipid compositions. (**A**) Schematic diagram of the molar ratios of the lipids used for the preparation of A_2A_AR in rHDLs. (**B**) [^35^S]-GTPγS binding to complexes of the purified G protein and A_2A_AR in rHDL(POPC/POPG), A_2A_AR in rHDL(ARA), and A_2A_AR in rHDL(DHA) in the presence of the full agonist. Results are expressed as the initial rates of the production of the GTPγS and G protein complex. Data are the means ± SEM of 3 (ARA), 13 (DHA), and 17 (POPC/POPG) experiments. *P < 0.0001 by unpaired two-tailed Student’s *t* test.

To investigate the effects of the DHA chains on the conformation and signaling activities of A_2A_AR, A_2A_AR was reconstituted into rHDLs composed of lipids with the DHA or ARA chains. Here, rHDLs containing the DHA and ARA chains are referred to as rHDL(DHA) and rHDL(ARA), respectively. Lipids containing 1-stearoyl-2-docosahexaenoyl-phosphocholine (SDPC), 1-stearoyl-2-docosahexaenoyl-phosphoglycerol (SDPG), and 1-stearoyl-2-arachidonoyl-phosphocholine (SAPC), in which one of the acyl chains is derived from DHA or ARA, were used for the preparation of A_2A_AR in rHDL(DHA) and A_2A_AR in rHDL(ARA) (fig. S6A). Considering the fact that more than 40% of the phospholipid acyl chains in cellular membranes are composed of those derived from DHA or ARA (*26*), we prepared A_2A_AR in rHDL(DHA) and A_2A_AR in rHDL(ARA) using lipids with SDPC:SDPG:POPC:POPG = 9:3:3:1 and SAPC:POPG = 3:1, respectively, in which 40% of the total acyl chains are derived from DHA or ARA (Fig. 2A and fig. S6A). The populations of the anionic lipids (SDPG, SAPG, and POPG) of A_2A_AR in rHDL(DHA) and A_2A_AR in rHDL(ARA) were the same as those of A_2A_AR in rHDL(POPC/POPG) (Fig. 2A and fig. S6A).

We used NMR to examine the lipid composition of A_2A_AR in rHDLs. In the ^1^H-1D spectrum of A_2A_AR in rHDL(DHA), an ensemble of resonances from the methyl groups of the DHA chains and another ensemble of resonances from the methyl groups of the other acyl chains were observed with 4:6 signal intensity ratio, suggesting that DHA constitutes 40% of the acyl chains in A_2A_AR in rHDL(DHA) (fig. S6B). In the ^1^H-1D spectrum of A_2A_AR in rHDL(ARA), a resonance from the methyl groups of the polar head of SAPC, an ensemble of resonances from methylene groups connecting the C═C double bonds in the ARA chain, the methylene groups farther away from the C═C double bonds, and the methyl groups of the acyl chains were observed (fig. S6C). The signal intensities of these resonances indicate that ARA constitutes ~35% of the fatty acids in A_2A_AR in rHDL(ARA). Therefore, the populations of DHA and ARA in the acyl chains are almost identical to those of the lipids used for the preparation of the rHDLs. In the ^31^P-1D spectrum of A_2A_AR in rHDL(POPC/POPG) and A_2A_AR in rHDL(DHA), the resonances from phosphatidylcholine and phosphoatidylglycerol were observed with a 3:1 signal intensity ratio (fig. S6D), suggesting that the population of the polar head groups is comparable to those of the lipids used for the preparation of the rHDLs. In the tritium-labeled full agonist binding assays, A_2A_AR in rHDL(DHA) and A_2A_AR in rHDL(ARA) exhibited almost identical dissociation constants (150 and 140 nM, respectively; fig. S6, E and F).

We examined the time course of the [^35^S]-GTPγS binding to the complex of A_2A_AR in rHDL(DHA), with the heterotrimeric G protein and the full agonist. As a result, the initial [^35^S]-GTPγS binding rate for A_2A_AR in rHDL(DHA) was 1.88 ± 0.07 times higher than that for A_2A_AR in rHDL(POPC/POPG) (Fig. 2B). In contrast, the initial [^35^S]-GTPγS binding rate for A_2A_AR in rHDL(ARA) was only 1.10 ± 0.07 times higher than that for A_2A_AR in rHDL(POPC/POPG) (Fig. 2B).

There are differences between rHDL(POPC/POPG), rHDL(ARA), and rHDL(DHA) in the hydrocarbon chain lengths of the sn-1 chains. To examine the effects of the differences between samples in terms of the hydrocarbon chain length of the sn-1 chains, we compared the rHDLs with identical lipid compositions, except for the substitution of the DHA chains with other acyl chains. We examined the time course of the [^35^S]-GTPγS binding to A_2A_AR in rHDLs using lipids with SDPC:POPG = 3:1, in which the lipid composition is identical to rHDL(ARA) except for the substitution of the DHA chains for the ARA chains (fig. S7A). As a result, the initial [^35^S]-GTPγS binding rate was higher than that for A_2A_AR in rHDL(ARA) (fig. S7B). We also confirmed that A_2A_AR in rHDLs using lipids with 1,2-didocosahexaenoyl-phosphatidylcholine (DDPC):POPC:1,2-didocosahexaenoyl-phosphatidylglycerol (DDPG):POPG = 9:15:3:5, in which the lipid composition is identical to rHDL(POPC/POPG) except for the substitution of the DHA chains for the palmitoyl and oleic acid chains (fig. S7C), exhibited a higher initial [^35^S]-GTPγS binding rate than A_2A_AR in rHDL(POPC/POPG) (fig. S7D).

### Effect of lipids on the conformational equilibrium of A_2A_AR in rHDLs

**Fig. 3:**
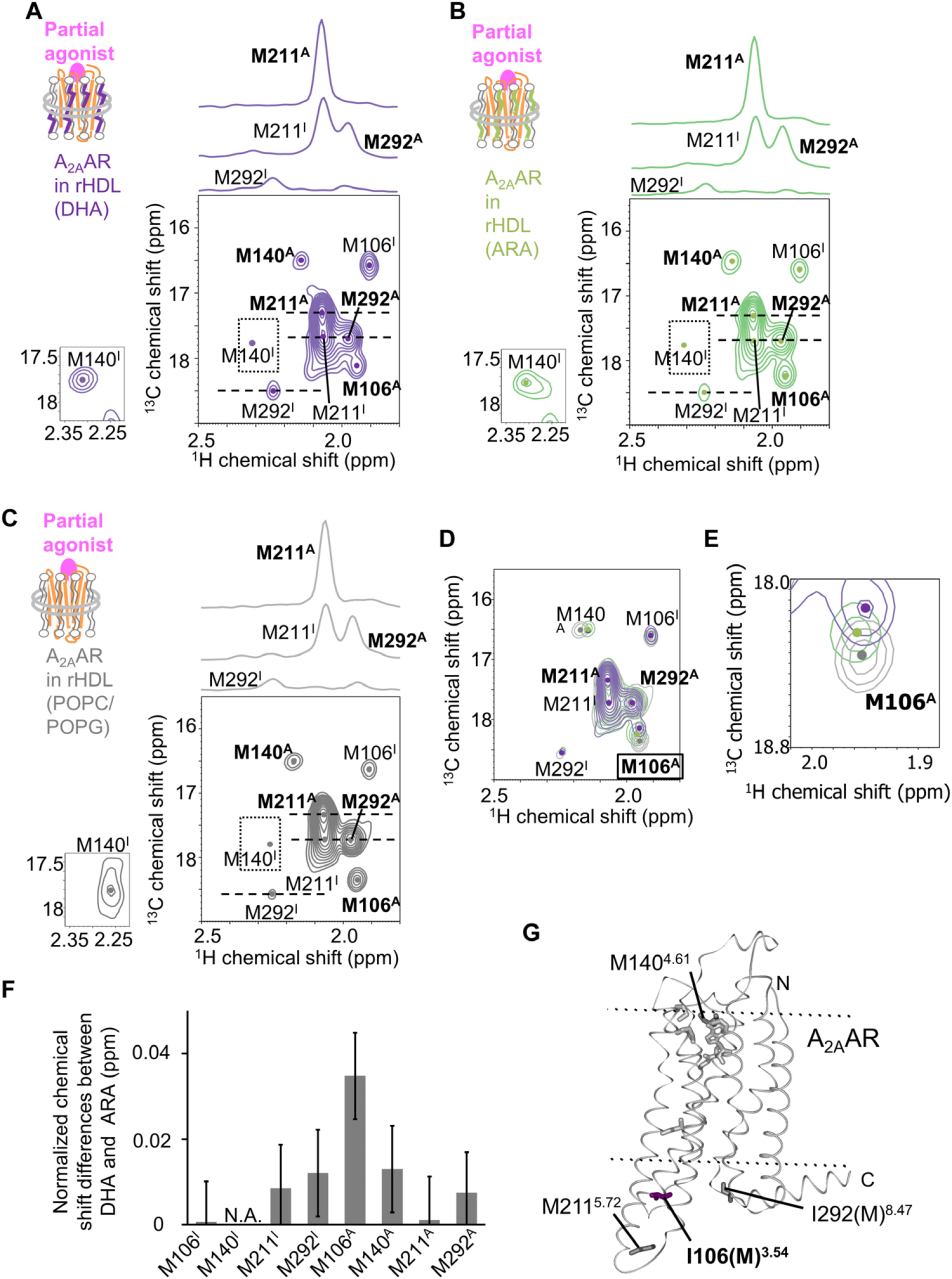
Conformations of A_2A_AR in rHDLs with various lipid compositions bound to the partial agonist. (**A** to **C**) ^1^H-^13^C HMQC spectra of [[α,β,β-^2^H, methyl-^13^C] Met, u-^2^H] A_2A_AR/I106M/I292M in rHDL(DHA) (A), in rHDL(ARA) (B), and in rHDL(POPC/POPG) (C) bound to the partial agonist. Cross-sections at the dashed lines are shown above each spectrum. The methionine methyl resonances are indicated, and the centers are indicated with dots. Superscripts I and A are added to the labels of the M106^3.54^, M140^4.61^, M211^5.72^, and M292^8.47^ signals with chemical shifts similar to those in the presence of the inverse agonist and the full agonist, respectively. The M140^I^ signals, which are lower than the minimum contour level, are enclosed in boxes and shown at the left of each spectrum with different contour levels. The methionine resonances are indicated, and the centers are indicated with dots. The spectra in (A), (B), and (C) are overlaid in (**D**) and (**E**). Only the region with M106^A^ signals is shown in (E). The spectrum in Fig. 3C is identical to that in Fig. 1H, except for the color. (**F**) Normalized chemical shift differences of the methionine methyl resonances between A_2A_AR in rHDL(POPC/POPG) and A_2A_AR in rHDL(DHA), Δδ, calculated by the equation Δδ = [(Δδ_1H_)^2^ + (Δδ_13C_/3.5)^2^]^0.5^, as reported previously (*36*). Error bars represent SDs of the chemical shifts of 100 synthetic time domain data from the in situ error analysis (*40*). (**G**) Distribution of the methionine residues in the crystal structure of A_2A_AR. The crystal structure of A_2A_AR with a full agonist, NECA (PDB code: 2YDV), is shown in ribbons in a side view, with the extracellular sides on the top. I106(M)^3.54^, which exhibited different chemical shifts between A_2A_AR in rHDL(DHA) and A_2A_AR in rHDL(ARA), is depicted by purple sticks. M140^4.61^, M211^5.72^, and I292(M)^8.47^, in which the chemical shifts of A_2A_AR in rHDL(DHA) were almost identical to those of A_2A_AR in rHDL(ARA), are depicted by dark gray sticks. M174^5.37^, M177^5.40^, M193^5.54^, and M270^7.34^, in which the resonances were not observed in the presence of the full agonist, are depicted by gray sticks. NECA is depicted by gray sticks. The membrane position generated by the OPM database (http://opm.phar.umich.edu/) is indicated.

To investigate the effects of the lipids on the conformational equilibrium of A_2A_AR in rHDLs, the ^1^H-^13^C HMQC spectra of A_2A_AR/I106M/I292M, labeled with [[α,β,β,-^2^H, methyl-^13^C] Met, u-^2^H] and embedded in rHDL(DHA) and rHDL(ARA), were observed in the presence of the partial agonist. In these spectra, M106^3.54^, M140^4.61^, M211^5.72^, and M292^8.47^ exhibited two resonances, and their chemical shifts were similar to those of the inactive (M106^I^, M140^I^, M211^I^, and M292^I^) and active (M106^A^, M140^A^, M211^A^, and M292^A^) states in rHDL(POPC/POPG) (Fig. 3, A to C). The intensity ratios of the active state signals, relative to the inactive ones, of A_2A_AR in rHDL(DHA) were less than 1.1-fold higher than those of rHDL(ARA), suggesting that the populations of the inactive and active states are not affected by the type of surrounding lipids, which cannot explain the 1.7-fold higher GDP/GTP reaction rate of A_2A_AR in rHDL(DHA) than A_2A_AR in rHDL(ARA) (Fig. 2B). In contrast, the M106^A^ signal of A_2A_AR in rHDL(ARA) exhibited a remarkably different chemical shift from that of A_2A_AR in rHDL(DHA) (Fig. 3, D and E). Comparisons of the M106^A^ signals of A_2A_AR in rHDL(DHA), rHDL(ARA), and rHDL(POPC/POPG) revealed that the M106^A^ signal of A_2A_AR in rHDL(ARA) was observed at a chemical shift in between those of A_2A_AR in rHDL(DHA) and A_2A_AR in rHDL(POPC/POPG) (Fig. 3, D to G), suggesting that, in the presence of the partial agonist, the amount of each substate in the active state of A_2A_AR is affected by the type of surrounding lipids.

**Fig. 4:**
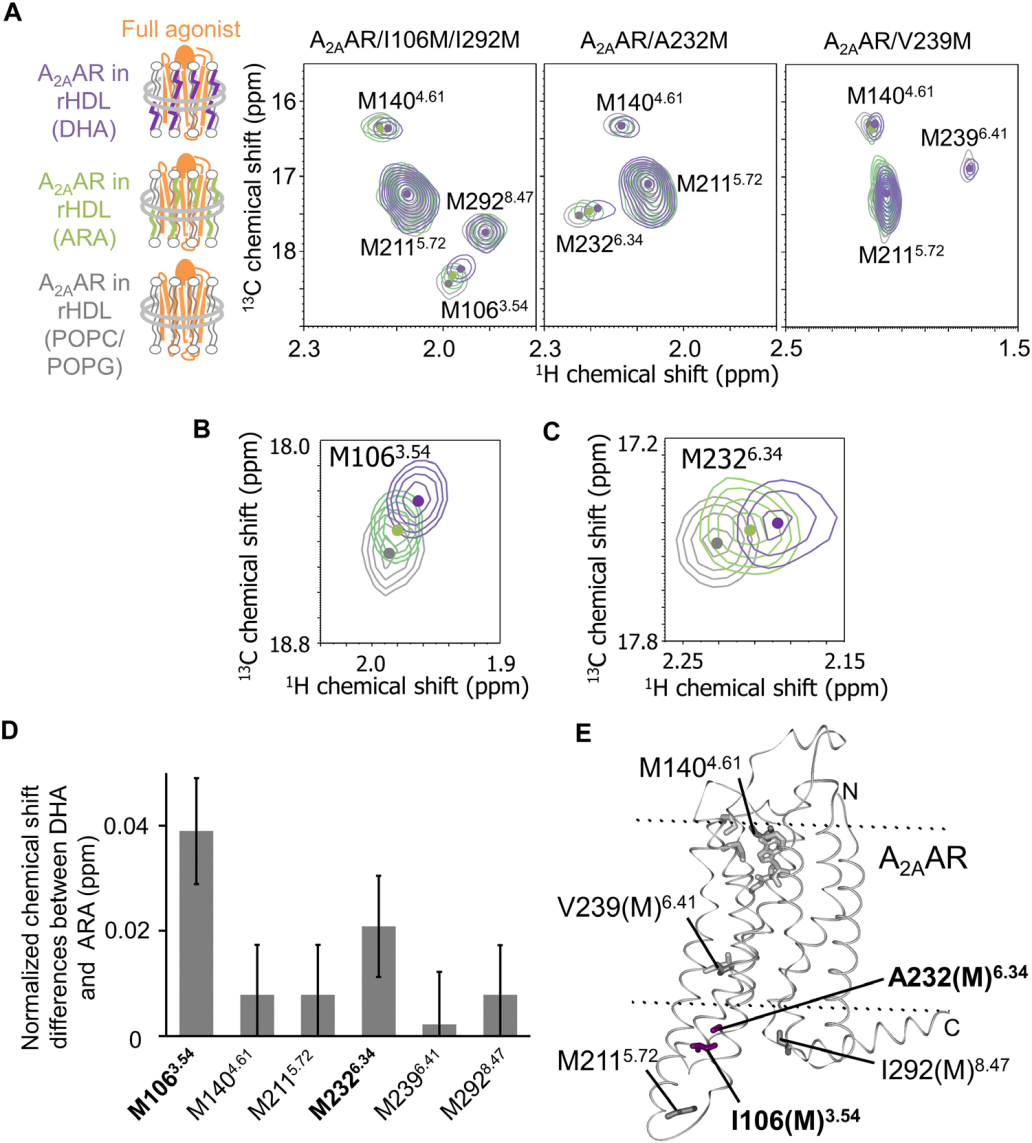
Conformations of A_2A_AR in rHDLs with various lipid compositions. (**A** to **C**) Overlaid ^1^H-^13^C HMQC spectra of A_2A_AR/I106M/I292M, A_2A_AR/A232M, and A_2A_AR/V239M, labeled with [[α,β,β-^2^H, methyl-^13^C] Met, u-^2^H] and embedded in rHDL(POPC/POPG) (gray), rHDL(ARA) (green), or rHDL(DHA) (purple). Only the regions with M106^3.54^ and M232^6.34^ methyl resonances are shown in (B) and (C), respectively. The methionine methyl resonances are indicated, and the centers are indicated with dots. (**D**) Normalized chemical shift differences of the methionine methyl resonances between A_2A_AR in rHDL(ARA) and A_2A_AR in rHDL(DHA) and the errors were calculated in manners similar to Fig. 3F. (**E**) Distribution of the methionine residues in the crystal structure of A_2A_AR. The crystal structure of A_2A_AR with a full agonist, NECA (PDB code: 2YDV), is shown in ribbons in a side view, with the extracellular sides on the top. I106(M)^3.54^ and A232(M)^6.34^, which exhibited different chemical shifts between A_2A_AR in rHDL(DHA) and A_2A_AR in rHDL(ARA), are depicted by purple sticks. M140^4.61^, M211^5.72^, V239(M)^6.41^, and I292(M)^8.47^, in which the chemical shifts of A_2A_AR in rHDL(DHA) were almost identical to those of A_2A_AR in rHDL(ARA), are depicted by dark gray sticks. M174^5.37^, M177^5.40^, M193^5.54^, and M270^7.34^, in which the resonances were not observed in the presence of the full agonist, are depicted by gray sticks. NECA is depicted by gray sticks. The membrane position generated by the OPM database (http://opm.phar.umich.edu/) is indicated.

To further investigate the effects of lipids on the equilibria among the multiple conformations in the active state of A_2A_AR, ^1^H-^13^C HMQC spectra of A_2A_AR/I106M/I292M, A_2A_AR/A232M, and A_2A_AR/V239M, labeled with [[α,β,β,-^2^H, methyl-^13^C] Met, u-^2^H] and embedded in rHDL(DHA) or rHDL(ARA), were observed in the presence of the full agonist (Fig. 4A). In the spectra, the resonances from M106^3.54^ and M232^6.34^ of A_2A_AR in rHDL(ARA) were markedly different from those of A_2A_AR in rHDL(DHA), and the chemical shifts of the M106^3.54^ and M232^6.34^ signals of A_2A_AR in rHDL(ARA) were in between those of A_2A_AR in rHDL(DHA) and A_2A_AR in rHDL(POPC/POPG) (Fig. 4, B and C). These residues exist at the cytoplasmic ends of TM3 and TM6 (Fig. 4E) and are >8 Å away from the lipid bilayers and thus would be less affected by direct interactions with lipids. The chemical shifts of the M140^4.61^, M211^5.72^, M239^6.41^, and M292^8.47^ signals, which are on the flexible loop region or farther away from the cytoplasmic ends of TM3 and TM6, were almost identical in these spectra (Fig. 4, A, D, and E).

For the analyses of the conformation of A_2A_AR, we observed the ^1^H-^13^C HMQC spectrum of the ternary complex, composed of A_2A_AR/A232M in rHDL(POPC/POPG), a full agonist, and a G protein mimetic. The crystal structures of A_2A_AR revealed that the G protein mimetic induces the large TM6 rotation (*7*). The comparison between the M232 signals of A_2A_AR in rHDL(POPC/POPG) and rHDL(DHA) in the presence of a full agonist and that of the ternary complex revealed that the M232 signal of A_2A_AR/A232M in rHDL(DHA) was observed in the middle of those of A_2A_AR/A232M in rHDL(POPC/POPG) in the absence and presence of the G protein mimetic (fig. S7E).

### Conformations of A_2A_AR in rHDL(DHA) and A_2A_AR in rHDL(ARA) investigated by solvent PRE experiments

**Fig. 5:**
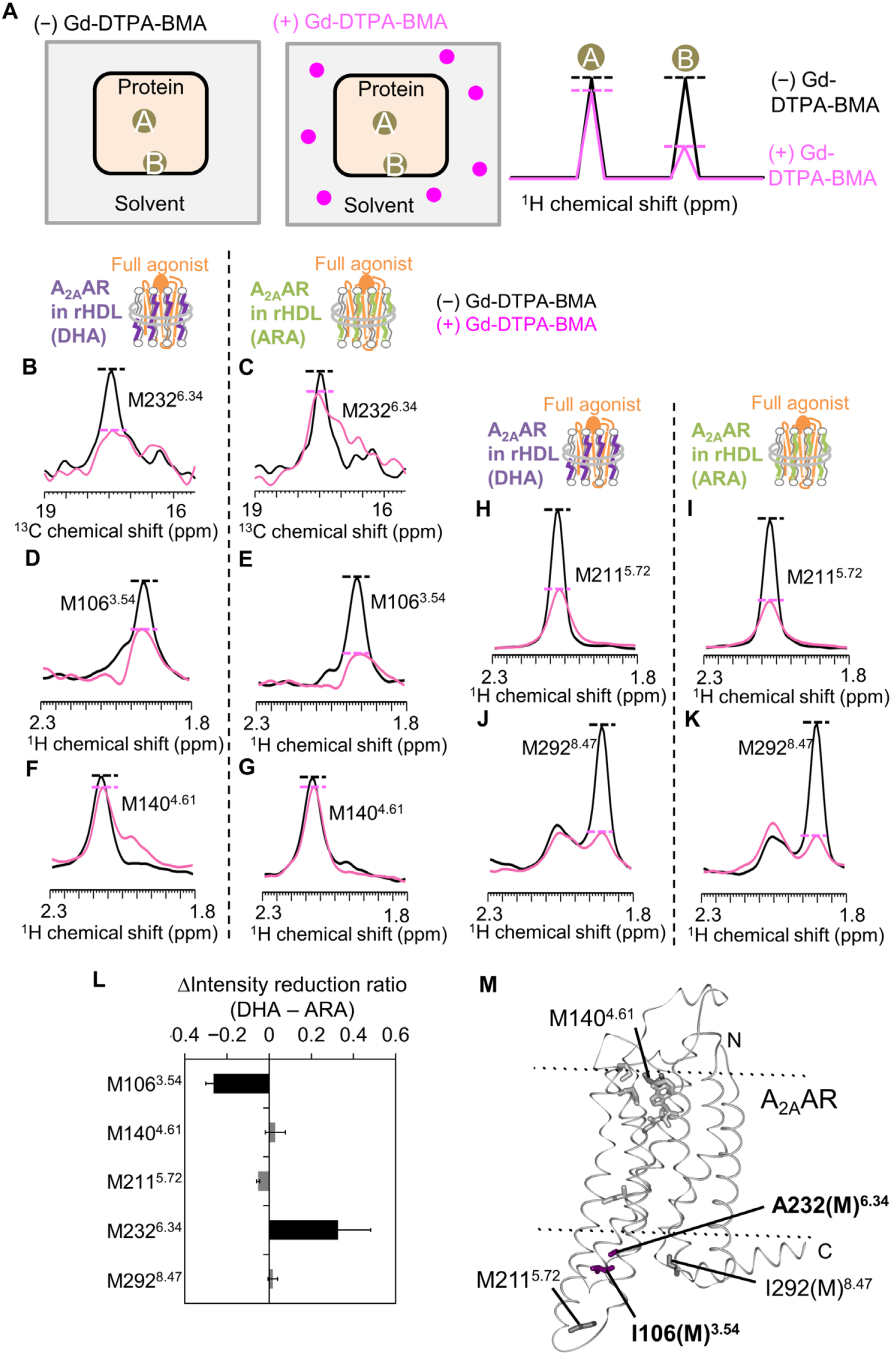
Conformations of A_2A_AR in rHDL(DHA) and A_2A_AR in rHDL(ARA) examined by solvent PRE experiments. (**A**) Schematic diagrams of the solvent PRE experiments. (**B** to **K**) Overlaid cross sections of the resonances from M232^6.34^, M106^3.54^, M140^4.61^, M211^5.72^, and M292^8.47^ in the ^1^H-^13^C HMQC spectra of A_2A_AR in rHDL(DHA) and A_2A_AR in rHDL(ARA), without Gd-DTPA-BMA (black) and with 5 mM Gd-DTPA-BMA (magenta). (**L**) Plots of the differences between the intensity reduction ratios observed for A_2A_AR in rHDL(DHA) and those observed for A_2A_AR in rHDL(ARA). The error bars represent the experimental errors, calculated from the root sum squares of the (noise level/signal intensity) in the four spectra, with and without Gd-DTPA-BMA in the experiments using A_2A_AR in rHDL(DHA) and A_2A_AR in rHDL(ARA). (**M**) Distribution of the methionine residues in the crystal structure of A_2A_AR. The crystal structure of A_2A_AR with a full agonist, NECA (PDB code: 2YDV), is shown in ribbons in a side view, with the extracellular sides on the top. I106(M)^3.54^ and A232(M)^6.34^, which exhibited different solvent accessibilities between A_2A_AR in rHDL(DHA) and A_2A_AR in rHDL(ARA), are depicted by purple sticks. M140^4.61^, M211^5.72^, and I292(M)^8.47^, in which the solvent accessibilities of A_2A_AR in rHDL(DHA) were almost identical to those of A_2A_AR in rHDL(ARA), are depicted by dark gray sticks. M174^5.37^, M177^5.40^, M193^5.54^, and M270^7.34^, with resonances that were not observed in the presence of the full agonist, are depicted by gray sticks. NECA is depicted by gray sticks. The membrane position generated by the OPM database (http://opm.phar.umich.edu/) is indicated.

To examine the structural characteristics of A_2A_ARs in rHDL(DHA) and rHDL(ARA), the solvent accessibilities of the methionine residues were examined by solvent paramagnetic relaxation enhancement (PRE) experiments. In these experiments, Gd-diethylenetriamine pentaacetic acid-bismethylamide (Gd-DTPA-BMA), a highly water-soluble paramagnetic probe that lacks a site for protein binding (*27*), was added to the sample containing A_2A_AR in rHDL (Fig. 5A). The PRE arising from the magnetic dipolar interactions between the nuclear spin and the unpaired electron spin of the diffusing paramagnetic probe enhances the relaxation of the nuclear spins in a distance-dependent manner, leading to the intensity reduction of the NMR signals of the solvent-exposed residues (Fig. 5A). In the ^1^H-^13^C HMQC spectrum of A_2A_AR in rHDL(DHA) in the presence of the full agonist and Gd-DTPA-BMA, the M232^6.34^ signal intensity was remarkably lower than that in the absence of Gd-DTPA-BMA (Fig. 5B). In the case of A_2A_AR in rHDL(DHA), the M232^6.34^ signal intensity was more affected by Gd-DTPA-BMA than that of A_2A_AR in rHDL(ARA) (Fig. 5C), suggesting that M232^6.34^ is more accessible to the solvent in rHDL(DHA) than in rHDL(ARA). In contrast, the M106^3.54^ signal intensity of A_2A_AR in rHDL(DHA) was less affected by Gd-DTPA-BMA than that of A_2A_AR in rHDL(ARA) (Fig. 5, D and E), suggesting that M106^3.54^ has lower solvent accessibility in rHDL(DHA) than in rHDL(ARA). The Gd-DTPA-BMA–induced intensity reductions observed for the M140^4.61^, M211^5.72^, and M292^8.47^ signals of A_2A_AR in rHDL(DHA) were almost identical to those of A_2A_AR in rHDL(ARA) (Fig. 5, F to M).

## DISCUSSION

### Effects of the DHA chains on the function-related dynamics of A_2A_AR

Our NMR experiments with A_2A_AR in rHDLs revealed that A_2A_AR, in the presence of the partial agonist, exists in equilibrium between the active state, which includes multiple substates, and the inactive state (fig. S5, G to J) and that the full agonist skews the populations of these states toward the active state (fig. S5, A, B, and F). In addition, the DHA chains induced the distribution change in the multiple active substates of A_2A_AR bound to the partial agonist and full agonist (Figs. 3 and 4). These effects of lipids on the conformational equilibria would not be observed in A_2A_AR experiments under conditions using micelles, thus highlighting the importance of the utilization of rHDLs in this study.

On the basis of the comparison between the A_2A_AR crystal structures in the forms bound with an inverse agonist and in the ternary complex with a full agonist and a G protein mimetic, the following structural mechanism for the A_2A_AR activation was proposed (fig. S8) (*7*–*9*). The full agonist directly induces the movements of H250^6.52^, T88^3.36^, W246^6.48^, S277^7.42^, and H278^7.43^, which, in turn, induce the repositioning of V186^5.47^, P189^5.50^, I92^3.40^, F242^6.44^, and N280^7.45^, along with the movements of the intracellular sides of TM3, TM5, TM6, and TM7 (fig. S8). The movement of TM6 requires the reorganization of the side chains of Y197^5.58^ and Y288^7.53^. Y288^7.53^ constitutes a highly conserved NPxxY motif with N284^7.49^ and P285^7.50^, and the side-chain rotamer of Y288^7.53^ determines the activation state of the NPxxY motif (fig. S8) (*28*). The reorganization of the Y288^7.53^ side chain allows R102^3.50^ to adopt a fully extended conformation, packing against the side chain of Y391 in the α5 helix of the engineered G protein (fig. S8). Therefore, the side-chain rotamer of Y288^7.53^ is sensitive to the conformational change upon A_2A_AR activation. The ^1^H chemical shift of the M292^8.47^ methyl signals depends on the ring current effects from the neighboring aromatic ring of Y288^7.53^. The ^1^H chemical shifts of M292^8.47^ in the presence of the inverse agonist [2.23 parts per million (ppm)] exhibited a larger downfield ring current shift than that in the presence of the full agonist (1.9 ppm) (Fig. 1G and fig. S4, C and G). In the crystal structures of A_2A_AR in the inverse agonist-bound form, the ring current from Y288^7.53^ should induce a downfield shift in the ^1^H methyl atoms of M292^8.47^, whereas Y288^7.53^ is further away from M292^8.47^ in the crystal structure of the ternary complex form with a full agonist and a G protein mimetic (fig. S8). These structural characteristics are in good agreement with the above-described conformations indicated by the ^1^H chemical shifts, suggesting that the inactive and active states correspond well to the states containing the inactive and active NPxxY motifs: NPxxYoff and NPxxYon, respectively.

**Fig. 6:**
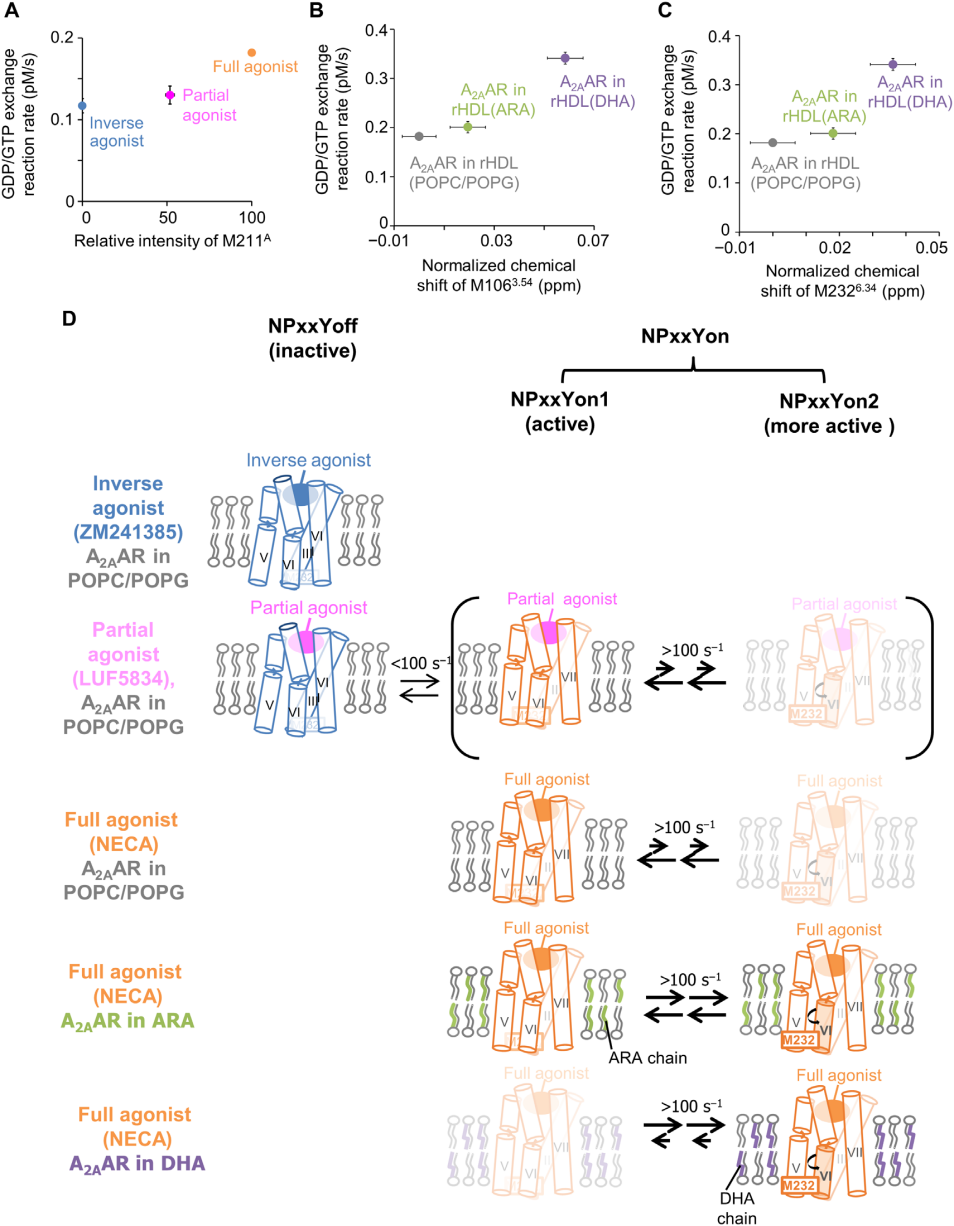
Signaling activity–related conformational equilibria of A_2A_AR. (**A** to **C**) Correlation between the population of each conformation in the equilibrium and the G protein signaling. Plots of the [^35^S]-GTPγS binding rate to the complexes of A_2A_AR in rHDLs and a heterotrimeric G protein, consisting of human Gα_s_, bovine Gβ_1_, and human Gγ_2_, with the full agonist versus the normalized intensity of the M211^A^ signal (A), and the normalized chemical shifts of M106^3.54^ (B) and M232^6.34^ (C). The [^35^S]-GTPγS binding rates and the errors are the same as those in Fig. 2B. The normalized chemical shifts and the errors were calculated in manners similar to those in Fig. 3F. (**D**) Schematic diagrams of the conformational equilibria of TM3, TM5, TM6, and TM7 of A_2A_AR in rHDLs. A_2A_AR in rHDL(POPC/POPG) bound to the inverse agonist-bound adopts an inactive NPxxYoff conformation. A_2A_AR in rHDL(POPC/POPG) bound to the partial agonist exists in equilibria between the NPxxYoff and multiple NPxxYon conformations, including the NPxxYon1 and NPxxYon2 conformations. The full agonist drives the equilibrium toward the NPxxYon conformations. The DHA chains redistribute the NPxxYon1 and NPxxYon2 conformations toward the NPxxYon2 conformation, which exhibits a large TM6 rotation angle and is more preferable for the G protein activation, as compared to the NPxxYon1 conformation.

The relative intensity of the M211^A^ signal exhibited a strong correlation with the G protein activation rates observed for A_2A_AR in the presence of the inverse agonist, partial agonist, and full agonist (Fig. 6A), suggesting that the population of the NPxxYon conformations in the equilibrium is closely related to the levels of the G protein signaling activities. Various class A GPCRs also exist in equilibria between the inactive and active states, and there are good correlations between the population of the active state and the ligand efficacies (*12*). We found that the DHA chains enhanced the G protein activation by A_2A_AR and that the acyl chains derived from DHA did not affect the populations of the NPxxYoff and NPxxYon states in the presence of the partial agonist (Fig. 3, A to C). In contrast, the DHA chain induced a distribution change among the multiple NPxxYon substates (Figs. 3, D and E, and 4). The chemical shifts of M106^3.54^ and M232^6.34^ correlated well with the G protein activation rates observed for A_2A_AR in rHDL(POPC/POPG), A_2A_AR in rHDL(ARA), and A_2A_AR in rHDL(DHA) (Fig. 6, B and C), suggesting that the NPxxYon state includes the substates that are more preferable for the G protein activation than the other NPxxYon substates. Here, the former and latter NPxxYon substates are referred to as the NPxxYon2 and NPxxYon1 substates, respectively.

Chemical shift differences among A_2A_AR-rHDL(DHA), A_2A_AR-rHDL(ARA), and A_2A_AR-rHDL(POPC/POPG) were observed for M106^3.54^ and M232^6.34^, which exist at the cytoplasmic ends of TM3 and TM6 of A_2A_AR (Figs. 3 and 4), suggesting that the structures of these regions are affected by the type of surrounding lipids. In addition, the solvent PRE experiments revealed that the DHA chains shift the equilibrium of A_2A_AR toward the conformations with the higher solvent accessibility of M232^6.34^ (Fig. 5). The crystal structures of A_2A_AR and other GPCRs indicated that the solvent accessibilities of the GPCR residues corresponding to M232^6.34^ of A_2A_AR reflect the rotational angle of TM6 (fig. S9, A and B, and see Supplementary Text). Our solvent PRE calculation of a model system, which exists in equilibrium between two conformations with different solvent accessibilities, suggested that the signal intensity reductions induced by Gd-DTPA-BMA, observed in the solvent PRE experiments, reflect the >30% population shift of the equilibrium among the conformations with >1 Å differences in the solvent accessibilities (fig. S9, C and D, and see Supplementary Text) (*27*). In addition, the M232^6.34^ signal of A_2A_AR/A232M in rHDL(DHA) was observed in the middle of those of A_2A_AR/A232M in rHDL(POPC/POPG) in the absence and presence of the G protein mimetic (fig. S7E). The crystal structures of A_2A_AR (*7*, *9*) revealed that the G protein mimetic induces large TM6 rotation by >25°. These results, along with the solvent PRE experiments, suggest that TM6 would undergo a large clockwise rotation, when viewed from the cytoplasmic surface, in the conformations in the NPxxYon2 substates (fig. S9, E and F, and see Supplementary Text). The TM6 rotation of A_2A_AR would facilitate the association between A_2A_AR and the G protein, which could be the rate-limiting step in the activation of the G protein (*29*), because the TM6 rotation is required for the van der Waals interactions between the residues in TM6 of a GPCR and the C-terminal helix of the G protein, formed in the crystal structures of GPCRs bound to both a full agonist and a trimeric G protein or its mimetics (*7*, *11*). Therefore, the NPxxYon2 conformation, which is highly populated in A_2A_AR-rHDL(DHA), would be preferable for the G protein activation.

On the basis of this structural interpretation of the resonances from M106^3.54^ and M232^6.34^, we propose the following signal regulation mechanism (Fig. 6D). A_2A_AR bound to the inverse agonist primarily adopts the inactive NPxxYoff conformation. In the presence of the full agonist, A_2A_AR exists in equilibria among the multiple active NPxxYon conformations, including the NPxxYon1 and NPxxYon2 conformations. The NPxxYon2 conformation adopts the large clockwise TM6 rotation and is more preferable for the G protein activation, as compared to the NPxxYon1 conformation. In the presence of the partial agonist, A_2A_AR exists in equilibrium between the aforementioned NPxxYoff and NPxxYon conformations. The DHA chains shift the equilibria among the NPxxYon conformations toward the NPxxYon2 conformation, which is more active than the NPxxYon1 conformation, leading to the enhanced G protein activation by A_2A_AR.

### Mechanism underlying the population shift of the equilibria of A_2A_AR by the DHA chains

Accumulating evidence suggests that the conformations and activities of membrane proteins can be modulated by their interactions with lipids, locations in membrane domains, and the physical properties of the lipid bilayer (e.g., lipid order, membrane fluidity, curvature stress, and hydrophobic mismatch) (*30*). The location in the domains can be excluded from the mechanism underlying the effects of DHA observed in this study, because the purified A_2A_AR in rHDLs is isolated from other proteins. Whereas the average area per lipid, bilayer thickness, lateral diffusion, and lateral pressure profiles of the lipid bilayer with the ARA chains are reportedly similar to those with the DHA chains (*31*), remarkable differences were successfully observed for the G protein activation rates of A_2A_AR in rHDL(DHA) and A_2A_AR in rHDL(ARA) in this study. We also confirmed that the G protein activation by A_2A_AR in rHDLs was not affected by the addition of trifluoroethanol, which reportedly perturbs the lateral pressure profiles (table S1) (*32*). Thus, the changes in the physical properties of the bilayer by the DHA chains would have a limited effect on the function-related dynamics of A_2A_AR. In the recent 15-μs all-atom molecular dynamics simulation of A_2A_AR, the local lipid environment around A_2A_AR was significantly enriched with lipids containing unsaturated chains (*33*). Thus, it is tempting to speculate that the interaction between A_2A_AR and the DHA chains facilitates the clockwise rotation of TM6.

### Relevance of stimulation of A_2A_AR by DHA

Although A_2A_AR in cells is embedded in membranes containing the DHA chains, information about the effects of DHA on the signaling activity of A_2A_AR was unavailable. In this study, the G protein activation rates of A_2A_AR in rHDL(DHA), A_2A_AR in rHDL(ARA), and A_2A_AR in rHDL(POPC/POPG) demonstrated that the DHA chains enhanced the G protein activation by A_2A_AR (Fig. 2B). In the lipid bilayers of the mammalian brain striatum, in which A_2A_AR is highly expressed (*1*), DHA constitutes 12 to 14% of the phospholipid acyl chains, and the population of the DHA chains is remarkably decreased to 3 to 8% by feeding DHA-deficient diets (*3*, *4*). In our experiment, the initial [^35^S]-GTPγS binding rate of A_2A_AR in rHDL(DHA), in which DHA constitutes 15% of the acyl chains, was 1.29 ± 0.03 times higher than that of A_2A_AR in rHDL(POPC/POPG) (*n* = 6). The effect of the DHA chains is higher than the level observed for the partial agonist (~10%; Fig. 1A), suggesting that the DHA chains in cell membranes play an indispensable role in the activation of A_2A_AR.

### Effects of various lipids on the conformation and activities of GPCRs

Cholesterol, anionic lipids, and other lipids, as well as the DHA chains, reportedly influence the GPCR activities (*21*). The compositions of biological membranes are affected by various pathological conditions, including aging, metabolic disease, Alzheimer’s disease, and cancer. Thus, knowledge about the mechanisms underlying the influences of lipids on the modulation of GPCR activities is helpful for understanding the GPCR functions under normal and pathological conditions. The effects of lipids on the signaling activities and the conformations of GPCRs may be different among various GPCRs and lipids, because the residues in the TM region directed toward the lipids are not conserved among class A GPCRs. For example, a previous ultraviolet–visible absorption spectroscopic study of rhodopsin indicated that the DHA chains facilitate the transition from metarhodopsin I to metarhodopsin II, which reportedly adopt conformations containing the inactive and active NPxxY motifs, respectively (*34*), whereas the populations of the NPxxYoff and NPxxYon conformations of A_2A_AR were not affected by the DHA chains (Fig. 3, A to C). The methyl resonances of deuterated GPCRs embedded in rHDLs with various lipid compositions are widely applicable for analyses of the effects of lipids on the function-related dynamics of GPCRs. Solid-state NMR has been used for studying lipid membrane protein interactions and conformations of membrane proteins embedded in lipid bilayer environments (*35*). Thus, solid-state NMR, which allows flexibility with respect to the lipid preparations, NMR detection schemes, and accessible temperature range, will be valuable for studying GPCRs in lipid bilayers.

Our studies using A_2A_AR in rHDL lipid bilayers revealed that the DHA chains enhanced the G protein activation by A_2A_AR. To our knowledge, this is the first report demonstrating the activation of A_2A_AR by DHA. In addition, our NMR studies of A_2A_AR in rHDL lipid bilayers revealed that A_2A_AR bound to the full agonist exists in equilibria among multiple NPxxYon conformations with faster exchange rates than the chemical shift difference, and DHA shifts the equilibria toward the conformations with a large clockwise rotation of TM6, which is preferable for the G protein activation. These findings provide insight into the physiological functions of A_2A_AR.

## MATERIALS AND METHODS

### Statistics and general methods

No statistical methods were used to predetermine the sample size. The experiments were not randomized. The investigators were not blinded to allocation during experiment and outcome assessment.

### Reagents

All reagents were purchased from Nacalai Tesque or Wako Pure Chemicals, unless otherwise noted. The [α,β,β-^2^H, methyl-^13^C]-l-methionine was synthesized by the enzymatic deuteration of [methyl-^13^C]-l-methionine (ISOTEC or Cambridge Isotope Laboratories) with *Escherichia coli* cystathionine γ-synthase, as previously described (*36*). The xanthine amine congener (XAC)–agarose gel was prepared as reported previously (*37*).

### Expression and purification of A_2A_AR

The complementary DNA fragment encoding human A_2A_AR (1 to 316), with a different secretion signal (α-factor) and a C-terminal 10× His-tag, was amplified by overlap extension polymerase chain reaction and cloned into the pPICZαA vector (Thermo Fisher Scientific) via the Eco RI–Sal I sites. Mutations were introduced with a QuikChange site-directed mutagenesis kit (Stratagene). The M1A/M4T mutation was introduced in all constructs, unless otherwise stated. The pPICZαA vector containing the A_2A_AR gene was linearized with Pme I and purified with a QIAquick nucleotide extraction kit (Qiagen). The linearized vector was transformed into *P. pastoris* X-33 cells by electroporation.

For the expression of [[α,β,β-^2^H, methyl-^13^C] Met, u-^2^H] A_2A_AR, the cells were initially grown in 10 ml of ^2^H-buffered minimal glycerol (BMG) medium [100 mM potassium phosphate (pH 6.0), 1.34% yeast nitrogen base, 4 × 10^−5^% biotin, 1% (v/v) glycerol, and H_2_O/D_2_O = 5/95], supplemented with zeocin (100 μg/ml), at 29°C for 2 to 3 days, with shaking at 230 revolutions per minute (rpm). The cells were then transferred to 1 liter of ^2^H-BMG medium, and the culture was incubated in a bioreactor vessel (Takasugi Co. Ltd., Tokyo, Japan) at 29°C, with shaking at 350 rpm. During this culture period, the pH value of the culture was maintained at 5.8 by adding a sodium hydroxide solution. After 2 days, 5 ml of glycerol was added, and the culture was continued for 26 hours. The cells were then centrifuged at 3000*g* and resuspended in ^2^H-buffered minimal methanol (BMM) medium [100 mM potassium phosphate (pH 6.0), 1.34% yeast nitrogen base, 4 × 10^−5^% biotin, 0.5% methanol-d_4_ (Cambridge Isotope Laboratories), and H_2_O/D_2_O = 5/95], supplemented with 10 mM theophylline, lysine (800 mg/liter), threonine (400 mg/liter), isoleucine (200 mg/liter, and [α,β,β-^2^H, methyl-^13^C] methionine (600 mg/liter). The cells were grown at 22°C, with shaking at 350 rpm for 20 hours, and 2.5 ml methanol-d_4_ was added. The incubation was continued for 40 hours, and the cells were harvested by centrifugation at 9000*g*.

All of the following procedures were either performed on ice or at 4°C. The cells were resuspended in buffer A [50 mM tris-HCl (pH 7.4), 200 mM NaCl, 5 mM EDTA, 0.2 mM 4-(2-aminoethyl)benzenesulfonyl fluoride hydrochloride, 20 μM leupeptin hemisulfate (Peptide Institute Inc.), 28 μM E-64, 0.6 μM aprotinin, and 30 μM pepstatin A (Peptide Institute Inc.)] and disrupted by agitation at 300 rpm at 4°C using 40 ml zirconia/silica beads (BioSpec Products). Glycerol was added to the cell lysate, at a final concentration of 10% (v/v). The cell lysate was centrifuged at 10,500*g* for 10 min, and the resulting supernatant was centrifuged at 140,000*g* for 60 min. The membrane pellet was suspended in 5 ml of buffer B [50 mM tris-HCl (pH 8.0), 200 mM NaCl, and 10% (v/v) glycerol], supplemented with 10 mM theophylline, and was stored at −80°C. The membranes were solubilized in 2 to 3 ml of buffer B, supplemented with 10% DDM and 10 mM theophylline, for 4 hours, and were then centrifuged at 150,000*g* for 30 min. The supernatant was batch incubated overnight with 1 ml of TALON metal affinity resin (Clontech). The resin was first washed with 15 ml of buffer B, supplemented with 0.05% DDM, 10 mM theophylline, and 5 mM imidazole, and subsequently washed with 15 ml of buffer B, supplemented with 0.05% DDM and 5 mM imidazole. The protein was eluted with 15 ml of buffer B, supplemented with 0.05% DDM and 250 mM imidazole. The eluate was batch incubated for 5 hours with 1 ml of XAC-agarose gel. The resin was washed with 5 ml of buffer B, supplemented with 0.05% DDM, and the protein was eluted with 16 ml of buffer B, supplemented with 0.05% DDM and 30 mM theophylline. The eluted A_2A_AR was concentrated to ~1 mg/ml, using a centrifugal filter device (Amicon Ultra-15, 30 kDa molecular weight cutoff, Millipore), and was stored at −80°C.

### Preparation of A_2A_AR in rHDLs

MSP1E3 was expressed and purified as previously described (*20*), with minor modifications, as follows. The double-stranded DNA encoding the N-terminal 7× His-tag, tobacco etch virus (TEV) protease recognition site, and MSP1E3 gene (*19*) was transferred into the pET43a vector (Novagen), with a modification of the nucleotides around the Psh AI site from 5′-GACAAGAGTCCGGGAGC-3′ to 5′-GACAAGTGTCCGGGCTTCTCCTCAACGATATCTGAGC-3′, using T4 DNA polymerase and T4 DNA ligase. The gene encoding the NUS tag was removed by Nde I digestion and subsequent ligation, and a gene encoding the C-terminal 1D4 tag was inserted with the QuikChange site-directed mutagenesis kit. The plasmid for MSP1 expression was constructed in a similar manner, except for the absence of the C-terminal 1D4 tag. The *E. coli* BL21 (DE3) cells were transformed with the plasmid and cultured in Terrific Broth medium at 37°C. Protein expression was induced by the addition of 1 mM isopropyl 1-thio-β-d-galactopyranoside, when the optical density at 600 nm (OD_600_) reached 2.5. After 3 hours of induction, the cells were harvested and sonicated in buffer containing 20 mM tris-HCl (pH 8.0), 300 mM NaCl, 0.1 mM 4-(2-aminoethyl)benzenesulfonamide, and protease inhibitor cocktail (Nacalai Tesque). The cell lysate was centrifuged at 20,000*g* for 30 min, and the precipitate was solubilized in buffer containing 1% (v/v) Triton X-100. After centrifugation at 100,000*g* for 1 hour, the supernatant was batch incubated for 2 hours with 10 ml of HIS-Select resin (Sigma-Aldrich). The resin was washed with 500 ml of buffer containing 1% (v/v) Triton X-100, followed by 500 ml of buffer containing 10 mM sodium cholate and 100 ml of buffer containing 10 mM imidazole. The protein was eluted by 50 ml of buffer containing 300 mM imidazole and was dialyzed against buffer containing 50 mM NaPi (pH 8.0), 200 mM NaCl, and 10% (v/v) glycerol. The His-tag was cleaved by TEV protease and removed by passage through HIS-Select resin.

Solutions of POPC, POPG, SDPC, SDPG, SAPC, DDPC, and DDPG in chloroform (Avanti Polar Lipids) were mixed at molar ratios of POPC:POPG = 3:1, SDPC:POPC:SDPG:POPG = 9:3:3:1, SAPC:POPG = 3:1, SDPC:POPG = 3:1, DDPC:POPG:DDPG:POPG = 9:15:3:5, and SDPC:POPC:SDPG:POPG = 9:21:3:7. The solvent was evaporated under a nitrogen atmosphere and dried in vacuo, to form a lipid film. The film was solubilized by the addition of buffer C [50 mM tris-HCl (pH 8.0) and 200 mM NaCl], supplemented with 200 mM sodium cholate, for a final lipid concentration of 50 mM.

All of the following procedures were performed either on ice or at 4°C. The purified A_2A_AR in DDM micelles (30 nmol), MSP1E3, and lipids were mixed at a final molar ratio of 1:10:120 in 1.5 ml of buffer C and incubated for 1 hour. To remove the detergents, 80% (w/v) of Bio-Beads SM-2 (Bio-Rad) was added to the solution and incubated for 3 hours with gentle mixing. The supernatant was batch incubated for 2 hours with 0.25 ml of TALON resin. The resin was washed with 1.25 ml of buffer C, supplemented with 5 mM imidazole. The A_2A_AR in rHDLs was eluted from the resin with 1.5 ml of buffer C, supplemented with 250 mM imidazole. The eluate was further purified by size exclusion chromatography on a Superdex 200 10/300 GL column, eluted with buffer C.

For the NMR experiments, the purified A_2A_AR in rHDLs was concentrated with a centrifugal filter device (Amicon Ultra-4, 30 kDa molecular weight cutoff, Millipore), with simultaneous buffer exchange to buffer D [20 mM sodium phosphate (pH 7.0) and H_2_O/D_2_O = 1/99], supplemented with and without the ligands [100 μM NECA, regadenoson, ZM241385, or 25 μM LUF5834 (Tocris)]. For the solvent PRE experiments, Gd-DTPA-BMA (Omniscan, Daiichi Sankyo) was added to the sample at a final concentration of 5 mM.

### Radioligand-binding assays

The NECA saturation binding assays were performed by incubating the purified A_2A_AR in rHDLs with five different concentrations of [^3^H]-NECA (PerkinElmer), between 1 nM and 0.5 μM, in 100 μl of buffer E [50 mM tris-HCl (pH 8.0)]. The competition binding assays between [^3^H]-NECA and LUF5834 were performed by incubating the purified A_2A_AR in rHDLs with 50 nM of [^3^H]-NECA and eight different concentrations of LUF5834, between 0.1 and 1 μM, in 100 μl of buffer E. The competition binding assays between the [^3^H]-NECA and the nonlabeled NECA were performed by incubating the purified A_2A_AR in rHDLs with 50 nM of [^3^H]-NECA and 12 different concentrations of NECA, between 0.1 and 10 mM, in 100 μl of buffer E. After a 1-hour incubation on ice, the solution was applied to a NAP-5 size exclusion column (GE Healthcare). The fractions containing A_2A_AR were mixed with Optiphase Supermix (PerkinElmer), and the radioactivity was detected with a liquid scintillation counter (MicroBeta2, PerkinElmer). Nonspecific binding was assessed by performing identical reactions with an excess amount of ZM241385.

The ZM241385 saturation binding assays were performed by incubations of the membrane precipitate containing expressed A_2A_AR with 0.3, 1, and 3 nM [^3^H]-ZM241385 (American Radiolabeled Chemicals) in 200 μl of buffer E. After a 1-hour incubation at 25°C on an orbital shaker (200 rpm), the solution was passed through a UniFilter-96 GF/C (PerkinElmer) by a cell harvester (PerkinElmer). After a 1-hour incubation at 50°C, MicroScint (PerkinElmer) was added to the filter, and the radioactivity was detected with a liquid scintillation counter.

### Preparation of the G protein and its mimetic

Human Gα_s_ was prepared as previously described (*18*), with slight modifications, as follows. Double-stranded DNA encoding the N-terminal hexahistidine tag, thrombin recognition site, and human Gα_s_ gene was transferred into the pET15b vector (Novagen). The *E. coli* BL21 (DE3) cells were transformed with the plasmid and cultured in LB medium at 37°C. Protein expression was induced by the addition of 1 mM isopropyl 1-thio-β-d-galactopyranoside, when the OD_600_ reached 0.6 to 0.7. The cells were cultivated at 18°C for 6 hours after induction. After centrifugation at 9000*g* for 10 min, the cells were resuspended in buffer B [20 mM tris-HCl (pH 8.0), 5 mM 2-mercaptoethanol, 0.1 mM phenylmethylsulfonyl fluoride (PMSF), 1 mM MgCl_2_, and 0.1 mM GDP] and stored at −80°C. The cells were lysed by sonication and centrifuged at 150,000*g* for 30 min. NaCl was added to the resulting supernatant at a final concentration of 100 mM, and the solution was batch incubated for 2 hours with 5 ml of cOmplete His-tag purification resin (Roche), equilibrated with buffer B. The resin was washed with 50 ml of buffer B containing 500 mM NaCl, followed by 100 ml of buffer B containing 100 mM NaCl and 20 mM imidazole. The protein was eluted by 100 ml of buffer B containing 100 mM NaCl and 150 mM imidazole. The eluate was applied to 1 ml of HiTrap Q HP (GE Healthcare), equilibrated with buffer B. The protein was eluted by a linear concentration gradient of NaCl, from 0 to 500 mM, and the eluate was concentrated with a centrifugal filter device (Amicon Ultra-15, 30 kDa molecular weight cutoff). The buffer was exchanged to 20 mM tris-HCl (pH 8.0), 2 mM MgCl_2_, 0.5 mM EDTA, 20 μM GDP, 0.1 mM PMSF, and 10% (v/v) glycerol, and the sample was stored at −80°C. The heterotrimeric G protein consisting of human Gα_s_, bovine Gβ_1_, and human Gγ_2_ was prepared, as previously described (*19*).

The G protein mimetic was prepared as previously, with modifications as follows. The DNA fragment encoding the engineered Gα_s_ gene (mini-Gs) was cloned into the above-described plasmid for the expression of MSP1, which contains the N-terminal 7× His-tag and the TEV protease recognition site, with an In-Fusion HD cloning kit (Clontech). The *E. coli* BL21 (DE3) codon plus RP cells were transformed with the plasmid and cultured in LB medium at 37°C. Protein expression was induced by the addition of 50 μM isopropyl 1-thio-β-d-galactopyranoside, when the OD_600_ reached 0.6 to 0.7. The cells were cultivated at 25°C for 20 hours after induction. After centrifugation at 9000*g* for 15 min, the cells were resuspended in a buffer containing 40 mM Hepes (pH 7.5), 100 mM NaCl, 10% glycerol, 10 mM imidazole, 5 mM MgCl_2_, 50 μM GDP, 1 mM 4-(2-aminoethyl)benzenesulfonyl fluoride hydrochloride, 1.5 μM pepstatin A, 20 μM leupeptin, and 100 μM dithiothreitol and stored at −80°C. The cells were lysed by sonication and centrifuged at 38,000*g* for 45 min. The supernatant was added to the 10 ml of HIS-Select resin, and the flow-through was collected and added to the column. The column was washed with 200 ml of buffer G [20 mM Hepes (pH 7.5), 10% glycerol, 1 mM MgCl_2_, and 50 μM GDP] containing 40 mM imidazole and 500 mM NaCl, followed by 100 ml of buffer G containing 40 mM imidazole and 100 mM NaCl. The protein was eluted by 30 ml of a buffer G containing 500 mM imidazole and 100 mM NaCl. TEV protease was added to a final ratio of 1:20 w/w (TEV:mini-Gs) and incubated for 18 hours at room temperature. The sample was dialysed overnight against 2 liters of buffer G containing 100 mM NaCl and added to the 8 ml of HIS-Select resin. The flow-through was collected and passed through the column five times and was concentrated with a centrifugal filter device (Amicon Ultra-15, 10 kDa molecular weight cutoff). The protein was further purified by size exclusion chromatography on a HiLoad 26/60 Superdex prep grade column and eluted with buffer containing 10 mM Hepes (pH 7.5), 100 mM NaCl, 10% glycerol, 1 mM MgCl_2_, 1 μM GDP, and 0.1 mM tris(2-carboxyethyl)phosphine.

### [^35^S]-GTPγS binding assays

A_2A_AR in rHDLs and the G protein were mixed at final concentrations of 20 and 50 nM, respectively, in 100 μl of buffer F [50 mM tris-HCl (pH 8.0), 5 mM MgCl_2_, and 1 μM GDP], supplemented with or without ligand (100 μM NECA, 100 μM regadenoson, 100 μM ZM241385, or 25 μM LUF5834). The GDP/GTPγS exchange was initiated by the addition of [^35^S]-GTPγS, at a final concentration of 10 nM. After a 30-min incubation at 25°C, the reaction mixture was applied to a NAP-5 size exclusion column. The fractions containing the G protein were mixed with Optiphase Supermix, and the radioactivity was detected with the liquid scintillation counter.

### NMR experiments

The ^1^H-^13^C HMQC and one-dimensional ^1^H spectra of A_2A_AR in rHDLs and in DDM micelles were recorded with a Bruker Avance 800 spectrometer equipped with a cryogenic probe. The one-dimensional ^31^P spectra of A_2A_AR in rHDLs were recorded with a Bruker Avance 400 spectrometer equipped with a broadband/^19^F observe probe. ^1^H-^13^C HMQC spectra were recorded for 15 to 40 μM [[α,β,β-^2^H, methyl-^13^C] Met, u-^2^H] A_2A_AR or its mutants, in rHDLs and in DDM micelles in buffer D. In the NMR experiments with A_2A_AR in DDM micelles, 0.19% DDM and 0.038% cholesteryl hemisuccinate were added to the samples. In the NMR experiments with A_2A_AR in the UK432097-, NECA-, regadenoson-, ZM241385-, and LUF5834-bound states, UK432097, NECA, regadenoson, ZM241385, and LUF5834 were added to the samples at final concentrations of 25, 100, 100, 100, and 25 μM, respectively. In the solvent PRE experiments, Gd-DTPA-BMA was added to the samples at a final concentration of 5 mM. In the NMR experiments of the ternary complex, the engineered G protein was added to the sample with 15 μM A_2A_AR/A232M in the NECA-bound state at a final concentration of 30 μM. The spectral widths were set to 12,800 Hz and 6400 to 7400 Hz for the ^1^H and ^13^C dimensions, respectively, and the interscan delays were set to 1 s. In total, 512 × 128 complex points were recorded, and 256 scans/free induction decay gave rise to an acquisition time of 20 to 23 hours for each spectrum. The spectra were referenced to 3-(trimethysilyl)-1-propanesulfonic acid sodium salt, in both the ^1^H and ^13^C dimensions. One-dimensional ^1^H NMR spectra were recorded with a repetition period of 1.0 s, with 8096 complex data points and a spectral width of 12,800 Hz. One-dimensional ^31^P NMR spectra were recorded with a repetition period of 1.0 s, with 2048 complex data points and a spectral width of 3240 Hz. The assignments of the resonances from the DHA and ARA chains were derived from the previous report (*38*). All of the spectra were processed and analyzed by the Topspin 2.1, 3.1, or 3.5 software (Bruker).

### In situ chemical shift error analysis

The calculations were performed by in-house developed programs, written in the Python 2.7 programming language, supplemented with the extension modules NumPy 1.15, SciPy 1.1, and Cython 0.29. Synthetic two-dimensional time domain data, composed of 100 synthetic signals with relaxation rates and signal-to-noise ratios similar to those of the observed resonances, were generated using in-house developed programs. The data were processed in the same manner as the observed data, and the SDs of the chemical shifts were calculated.

### GPCR structure database analysis

In total, 182 coordinate files of GPCR structures were obtained from Protein Data Bank (PDB), and the structures without residues corresponding to “5.63” or “6.27” in the GPCRdb numbering scheme (*25*) were excluded from the analysis, because the solvent accessibilities were overestimated for these structures. For the remaining 118 structures, the lipid bilayer was added by CHARMM-GUI Membrane Builder (*39*).

In each structure, hypothetical atoms with a 3.5-Å radius, corresponding to Gd-DTPA-DMA, were evenly placed at grid points spaced 1 Å surrounding GPCR, and the hypothetical atoms in which at least one of the GPCR and/or lipid atoms are within the sum of the radii were removed. The distances between the closest hypothetical atom and the Cβ atom of the residues corresponding to M106^3.54^ and M232^6.34^ of A_2A_AR were used for the calculation of the solvent PRE, based on the Hwang-Freed model, as reported previously (*27*). The full crowding effect was not implemented, considering the low concentration of A_2A_AR in rHDLs, whereas the correlated motion of Gd-DTPA-BMA and A_2A_AR in rHDLs were taken into account by setting the diffusion coefficient of Gd-DTPA-BMA equal to that of A_2A_AR in rHDL (approach B) (*27*). The hydrodynamic radius of A_2A_AR in rHDLs was set to 6 nm.

The TM3 to TM6 distances are the distances between the mean coordinates of the main-chain atoms of “3.49,” “3.50,” “3.51,” “3.52,” “3.53,” and “3.54” residues (GPCRdb numbering scheme) (*25*) and those of the “6.31,” “6.32,” “6.33,” “6.34,” “6.35,” and “6.36” residues. The TM6 rotation angles are the angles between the vectors connecting the mean coordinates of the main-chain atoms of the “1.36,” “1.37,” “1.38,” “1.39,” “1.40,” “1.41,” “2.57,” “2.58,” “2.59,” “2.60,” “2.61,” “4.53,” “4.54,” “4.55,” and “4.56” residues and those of the “1.52,” “1.53,” “1.54,” “1.55,” “1.56,” “2.40,” “2.41,” “2.42,” “2.43,” “2.44,” “4.44,” “4.45,” “4.46,” and “4.47” residues and the vectors connecting the mean coordinates of the Cα and Cβ atoms of the “6.34” residue.

## Supplementary Material

aay8544_SM.pdf

## SUPPLEMENTARY MATERIALS

Supplementary material for this article is available at http://advances.sciencemag.org/cgi/content/full/6/12/eaay8544/DC1 Supplementary Text Fig. S1. Characterization of A_2A_AR prepared in rHDLs. Fig. S2. Assignments of the resonances from the methionine residues of A_2A_AR. Fig. S3. Introduction of methionine residues in the cytoplasmic ends of the A_2A_AR TM regions. Fig. S4. Resonances from methionine residues at the cytoplasmic ends of the A_2A_AR TM region. Fig. S5. NMR spectra of A_2A_AR in rHDL(POPC/POPG) at various temperatures. Fig. S6. Lipids used for the reconstitution of A_2A_AR into rHDLs. Fig. S7. Signaling activity and conformation of A_2A_AR in rHDL. Fig. S8. Conformational changes in the TM region of A_2A_AR upon activation. Fig. S9. Conformation of TM6. Table S1. [^35^S]-GTPγS binding to complexes of purified G protein and A_2A_AR in rHDL(POPC/POPG) with 0, 0.5, 1, and 2 mol% trifluoroethanol (TFE), in the presence of the full agonist.

## Appendix

View/request a protocol for this paper from *Bio-protocol*.
