# Supplementary material for: Activation of adenosine A2A receptor by lipids from docosahexaenoic acid revealed by NMR
Source: Sci Adv. 2020 Mar 18;6(12):eaay8544. doi: 10.1126/sciadv.aay8544 (PMC7080496; doi:10.1126/sciadv.aay8544)
Supplement: aay8544_SM.pdf [file aay8544_SM.pdf]

Science Advances
AAAS

advances.sciencemag.org/cgi/content/full/6/12/eaay8544/DC1

Supplementary Materials for

Activation of adenosine A_{2A} receptor by lipids from docosahexaenoic acid revealed by NMR

Takuya Mizumura, Keita Kondo, Masatoshi Kurita, Yutaka Kofuku, Mei Natsume, Shunsuke Imai, Yutaro Shiraishi, Takumi Ueda, Ichio Shimada*

*Corresponding author. Email: shimada@iw-nmr.f.u-tokyo.ac.jp

Published 18 March 2020, *Sci. Adv.* **6**, eaay8544 (2020)
DOI: 10.1126/sciadv.aay8544

This PDF file includes:

Supplementary Text
Fig. S1. Characterization of A_{2A}AR prepared in rHDLs.
Fig. S2. Assignments of the resonances from the methionine residues of A_{2A}AR.
Fig. S3. Introduction of methionine residues in the cytoplasmic ends of the A_{2A}AR TM regions.
Fig. S4. Resonances from methionine residues at the cytoplasmic ends of the A_{2A}AR TM region.
Fig. S5. NMR spectra of A_{2A}AR in rHDL(POPC/POPG) at various temperatures.
Fig. S6. Lipids used for the reconstitution of A_{2A}AR into rHDLs.
Fig. S7. Signaling activity and conformation of A_{2A}AR in rHDL.
Fig. S8. Conformational changes in the TM region of A_{2A}AR upon activation.
Fig. S9. Conformation of TM6.
Table S1. [^{35}S]-GTPγS binding to complexes of purified G protein and A_{2A}AR in rHDL(POPC/POPG) with 0, 0.5, 1, and 2 mol% trifluoroethanol (TFE), in the presence of the full agonist.

Supplementary Text

Solvent accessibilities of M232$^{6.34}$ reflect the arrangement of the transmembrane helices of A_{2A}AR

In the crystal structures of GPCRs in which TM6 undergoes clockwise rotation, when viewed from the cytoplasmic surface (e.g.; the crystal structure of A_{2A}AR bound to both the full agonist and G protein mimetic), residue corresponding to M232$^{6.34}$ of A_{2A}AR is outwardly directed and exposed to the solvent, as compared with other GPCR structures (e.g.; the crystal structure of A_{2A}AR bound to the inverse agonist) (fig. S9A). Analyses of the 118 crystal structures of GPCRs manifested that the solvent accessibilities of the residues corresponding to M232$^{6.34}$ in A_{2A}AR correlate well with the rotational angle of TM6 (fig. S9B). Therefore, the solvent accessibility of M232$^{6.34}$ in A_{2A}AR reflects the rotational angle of TM6.

The solvent accessibilities of M106$^{3.54}$ are sensitive to the conformations of TM3, TM5, and TM6. The decreased solvent accessibility of M106$^{3.54}$ in A_{2A}AR in rHDL(DHA) can be explained by the repositioning of TM5.

Calculation of the solvent PRE effects

In the case of the equilibrium among conformations with different solvent accessibilities, with faster exchange rates than the chemical shift difference, the weighted average of the solvent PREs of the conformations is observed (*27*). To determine whether the solvent PRE is sensitive to the differences in the A_{2A}AR conformations between rHDL(DHA) and rHDL(ARA), we performed a solvent PRE simulation in which a molecule exists in equilibrium between two conformations with different solvent accessibilities, with exchange rates faster than the chemical shift differences (fig. S9C and D). In these calculations, the distances of the closest approaches between the paramagnetic centers and the observed atoms are set to 5–13 Å, considering the fact that, in the crystal structures of GPCRs, the distances of the closest approaches between the hypothetical Gd-DTPA-BMA atoms and the residues corresponding to M232$^{6.34}$ range from 5 to 13 Å. Our calculations revealed that the signal intensity reductions induced by Gd-DTPA-BMA are sensitive to the > 30% population shift of the equilibrium among the conformations with > 1 Å differences in the solvent accessibilities of the observed atoms (fig. S9D).

Chemical shifts of M232 reflect the rotation of TM6

The crystal structures of A_{2A}AR with a full agonist in the absence and presence of a G-protein mimetic indicate that the TM6 rotation induces the movement of M232$^{6.34}$, leading to the reorganization of the position of M232$^{6.34}$ relative to F201$^{5.62}$ (fig. S9E and F). The ^{1}H chemical shift of the M232$^{6.34}$ methyl signals depends on the ring current effects from the neighboring aromatic ring of F201$^{5.62}$. Thus, the ring current effects on M232$^{6.34}$ are sensitive to the rotation of TM6.

The resonance from M232$^{6.34}$ of A_{2A}AR in rHDL(POPC/POPG) with a full agonist exhibited a ^{1}H upfield shift upon binding to the G protein mimetic (fig. S7E). In the crystal structures in the absence and presence of the G-protein mimetic, the ring current from F201$^{5.62}$ should induce downfield and upfield shifts, respectively (fig. S9E and F). These characteristics of the structure are in good agreement with the above-described conformations indicated by the ^{1}H chemical shifts, suggesting that the ^{1}H upfield shift of

the M232$^{6.34}$ signal of A_{2A}AR in rHDL(POPC/POPG), induced by the G-protein mimetic, corresponds to the TM6 rotation in the crystal structures (figs. S7E and S9E and F).

The amplitude of the ^{1}H chemical shift difference between A_{2A}AR in rHDL(DHA) and rHDL(POPC/POPG) was more than 35% of that of the chemical shift perturbation induced by the G-protein mimetic (fig. S7E). These results, along with the solvent PRE experiments, suggest that TM6 undergoes a large rotation in the NPxxY2 conformation, which is highly populated in A_{2A}AR in rHDL(DHA).

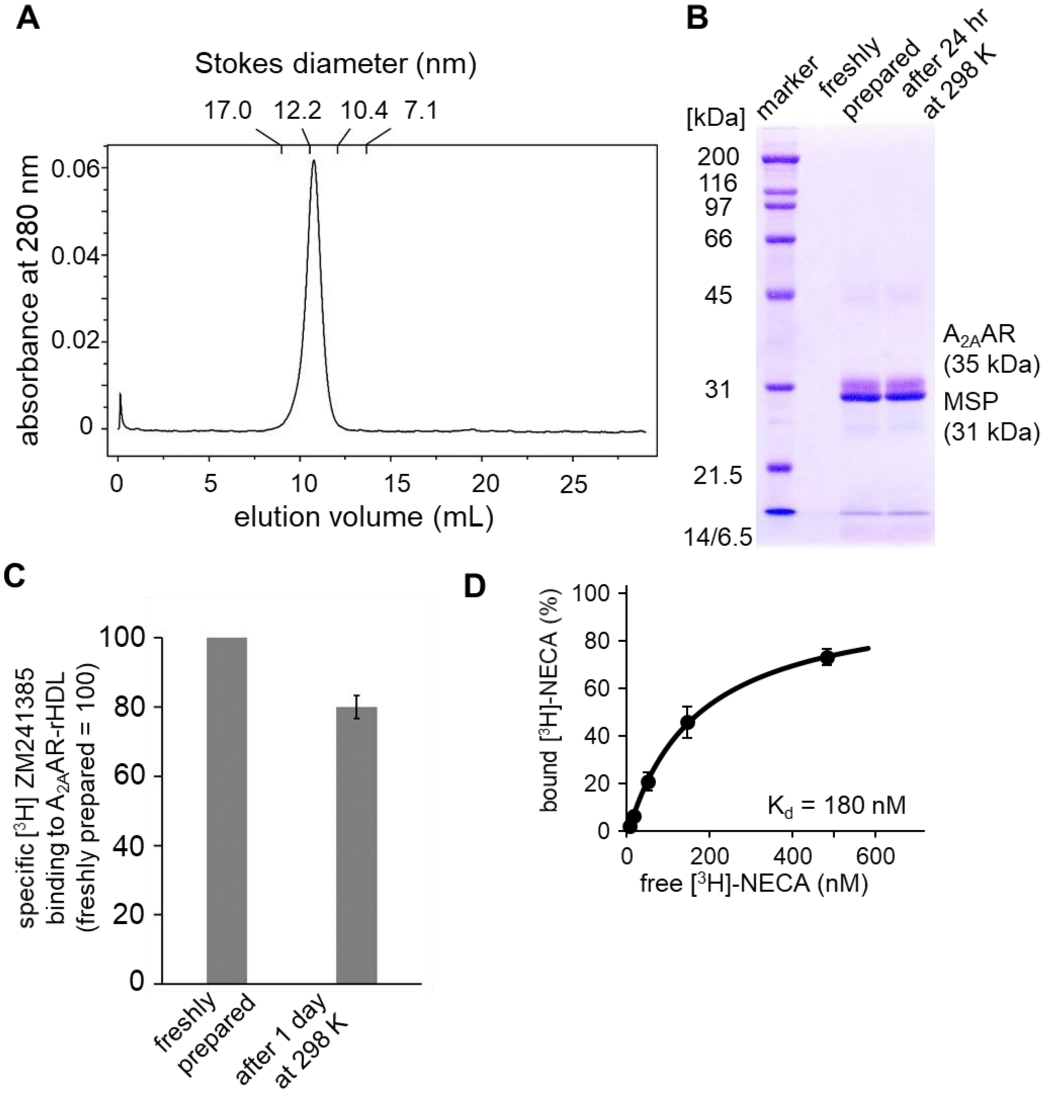

Fig. S1. Characterization of A_{2A}AR prepared in rHDLs. (A) Size exclusion chromatogram of purified A_{2A}AR in rHDL(POPC/POPG). Elution volumes corresponding to 17.0, 12.2, 10.4, and 7.1 nm Stokes diameters were determined by thyroglobulin, ferritin, catalase, and bovine serum albumin, respectively. **(B)** SDS-PAGE analyses of freshly prepared A_{2A}AR in rHDL(POPC/POPG) and that after 24 hr at 298 K. The samples were analyzed by 12% SDS-PAGE with Coomasie Brilliant Blue staining. **(C)** Amounts of A_{2A}AR in rHDL(POPC/POPG) that retained the [^{3}H]-ZM241385 binding activity before and after 1 day incubation at 298 K in the absence of the ligand. Specific binding of [^{3}H]-ZM241385 to A_{2A}AR was measured from the differences in the counts

with and without unlabeled ZM241385. **(D)** Measurement of [^{3}H]-NECA (full agonist) saturation binding to A_{2A}AR in rHDL(POPC/POPG). Data in (C) and (D) represent the mean ± standard error of the mean of six and four experiments, respectively.

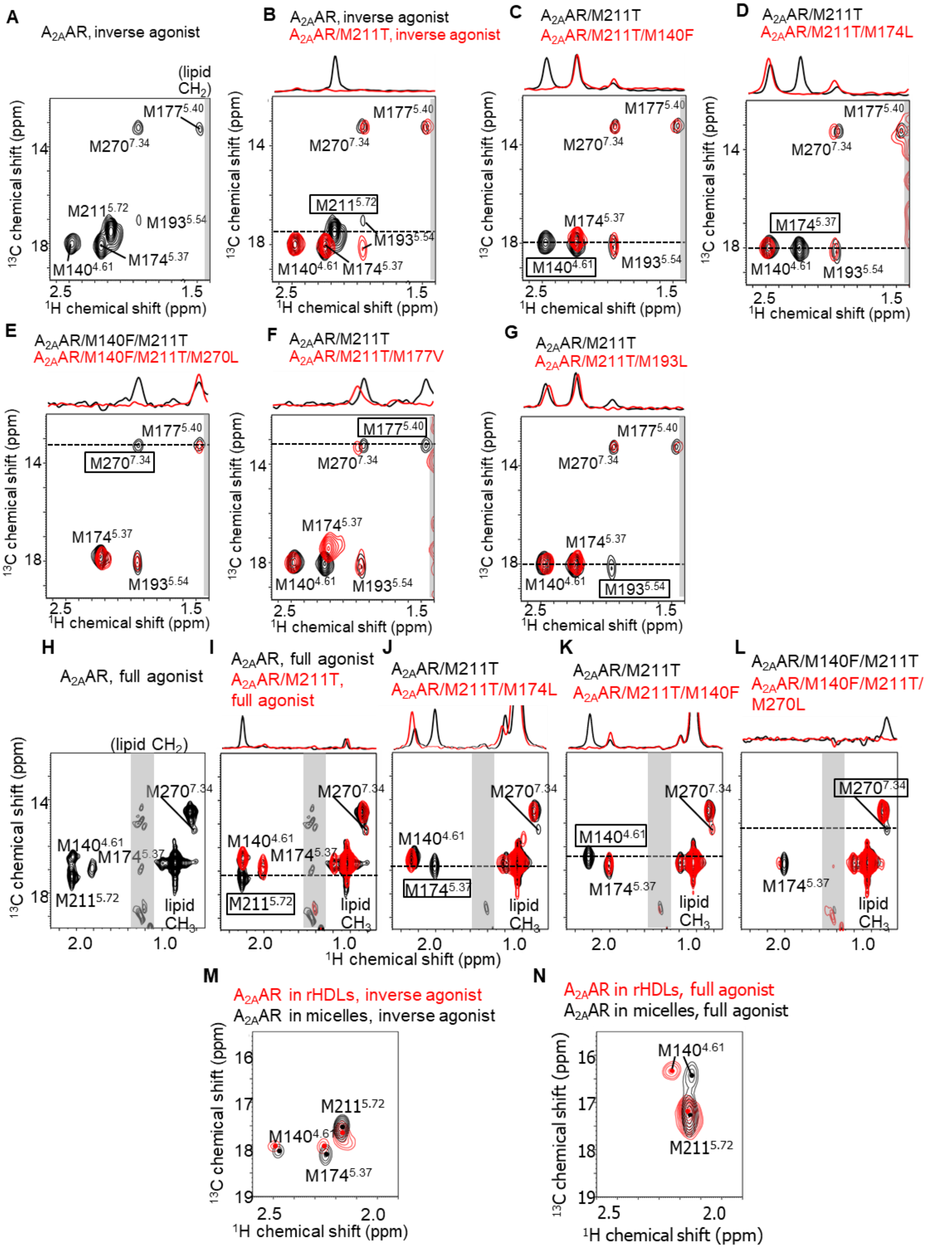

Fig. S2. Assignments of the resonances from the methionine residues of A_{2A}AR. (A) ^{1}H-^{13}C HMQC spectra of [[α,β,β-^{2}H, methyl-^{13}C] Met, u-^{2}H] A_{2A}AR bound to the inverse agonist (ZM241385) in DDM micelles. **(B)–(G)** Assignments of the resonances from M211$^{5.72}$ (B), M140$^{4.61}$ (C), M174$^{5.37}$ (D), M270$^{7.34}$ (E), M177$^{5.40}$ (F), and M193$^{5.54}$ (G) in

the presence of the inverse agonist. **(H)** ^{1}H-^{13}C HMQC spectra of [[α,β,β-^{2}H, methyl-^{13}C] Met, u-^{2}H] $A_{2A}AR$ bound to the full agonist in DDM micelles. Resonances from $M177^{5.40}$ and $M193^{5.54}$ were not observed, probably due to the line broadening of the resonances from these residues. **(I)–(L)**. Assignments of the resonances from $M211^{5.72}$ (I), $M140^{4.61}$ (J), $M174^{5.37}$ (K), and $M270^{7.34}$ (L) in the presence of the full agonist. **(M)** Overlaid ^{1}H-^{13}C HMQC spectra of [[α,β,β-^{2}H, methyl-^{13}C] Met, u-^{2}H] $A_{2A}AR$ bound to the full agonist in rHDLs (red) and that in DDM micelles (black). **(N)** Overlaid ^{1}H-^{13}C HMQC spectra of [[α,β,β-^{2}H, methyl-^{13}C] Met, u-^{2}H] $A_{2A}AR$ bound to the full agonist in rHDLs (red) and that in DDM micelles (black). The regions with methionine methyl resonances are shown, and the assigned resonances are indicated. In (B)–(G) and (I)-(L), black and red spectra represent the ^{1}H-^{13}C HMQC spectra of [[α,β,β-^{2}H, methyl-^{13}C] Met, u-^{2}H] $A_{2A}AR$ in DDM micelles with and without methionines to be assigned, respectively, and the mutants utilized in the assignments are indicated at the top of each panel. Cross-sections at the dashed grey lines are shown above each spectrum. The resonances from $M174^{5.37}$ and $M270^{7.34}$ in (M) and those from $M177^{5.40}$, $M193^{5.54}$, and $M270^{7.34}$ in (N), which are weak in the spectra of $A_{2A}AR$ in the DDM micelles, were not observed in the spectra of $A_{2A}AR$ in rHDLs, possibly due to its large size. The chemical shift differences between $A_{2A}AR$ in DDM micelles and rHDLs, observed for the resonances of $M140^{4.61}$ and $M174^{5.37}$ in (M) and those of $M140^{4.61}$ in (N), would be due to the interactions with lipids or DDM micelles, considering the fact that these residues are exposed to the lipids in rHDLs and DDM in micelles.

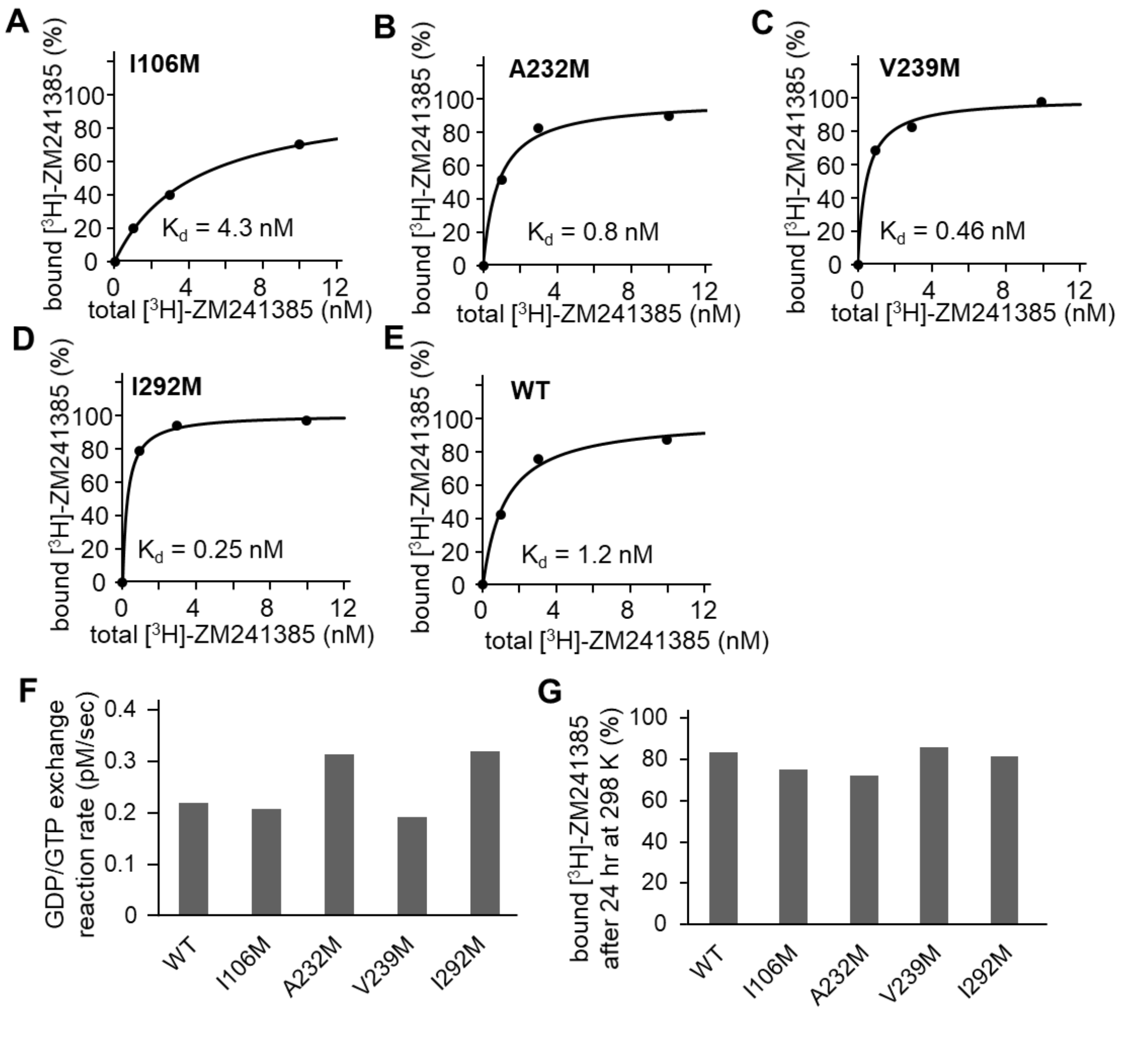

Fig. S3. Introduction of methionine residues in the cytoplasmic ends of the A_{2A}AR TM regions. (A)–(E) Saturation curves for the full agonist [^{3}H]-NECA binding to A_{2A}AR/I106M (A), A_{2A}AR/A232M (B), A_{2A}AR/V239M (C), A_{2A}AR/I292M (D), and A_{2A}AR (E) on the cell membrane. The dissociation constants of A_{2A}AR/I106M, which exhibited the reduced affinity for NECA, indicate that > 99% of the mutants bind to the ligands in the NMR samples. **(F)** [^{35}S]-GTPγS binding to complexes of purified G protein and A_{2A}AR in rHDL(POPC/POPG) in the presence of the full agonist. Results are expressed as the initial rates of the production of the GTPγS and G protein complexes. **(G)** Amounts of A_{2A}AR in rHDL(POPC/POPG) that retained the [^{3}H]-ZM241385 binding activity after a 24 hr incubation at 298 K in the absence of the ligand, relative to those before the incubation.

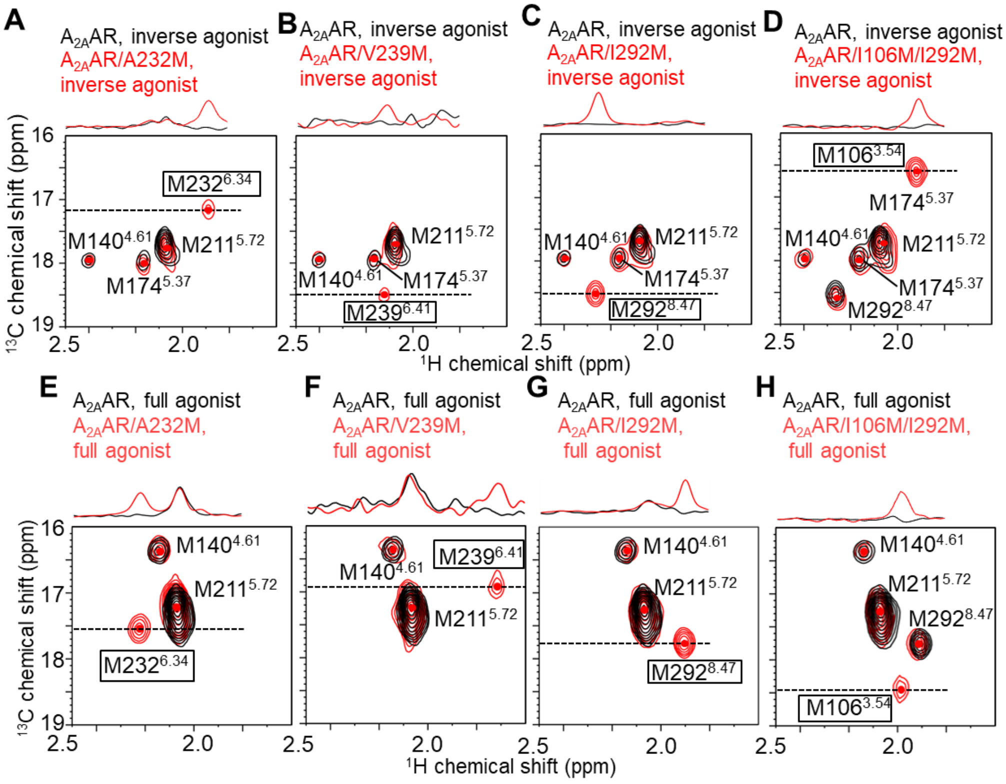

Fig. S4. Resonances from methionine residues at the cytoplasmic ends of the A_{2A}AR TM region. (A–D) NMR resonances from M232$^{6.34}$ (A), M239$^{6.41}$ (B), M292$^{8.47}$ (C), and M106$^{3.54}$ (D) of A_{2A}AR, bound to the inverse agonist in rHDLs. **(E)–(H)** NMR resonances from M232$^{6.34}$ (E), M239$^{6.41}$ (F), M292$^{8.47}$ (G), and M106$^{3.54}$ (H) of A_{2A}AR bound to the full agonist in rHDLs. The black and red spectra represent the ^{1}H-^{13}C HMQC spectra of [[α,β,β-^{2}H, methyl-^{13}C] Met, u-^{2}H] A_{2A}AR in rHDLs with and without the methionine residues, respectively. The mutants utilized in the assignments are indicated at the top of each panel. Only the regions with methionine methyl resonances are shown. The methionine methyl resonances are indicated, and the centers are indicated with dots. The cross-sections at the dashed grey lines are shown above each spectrum.

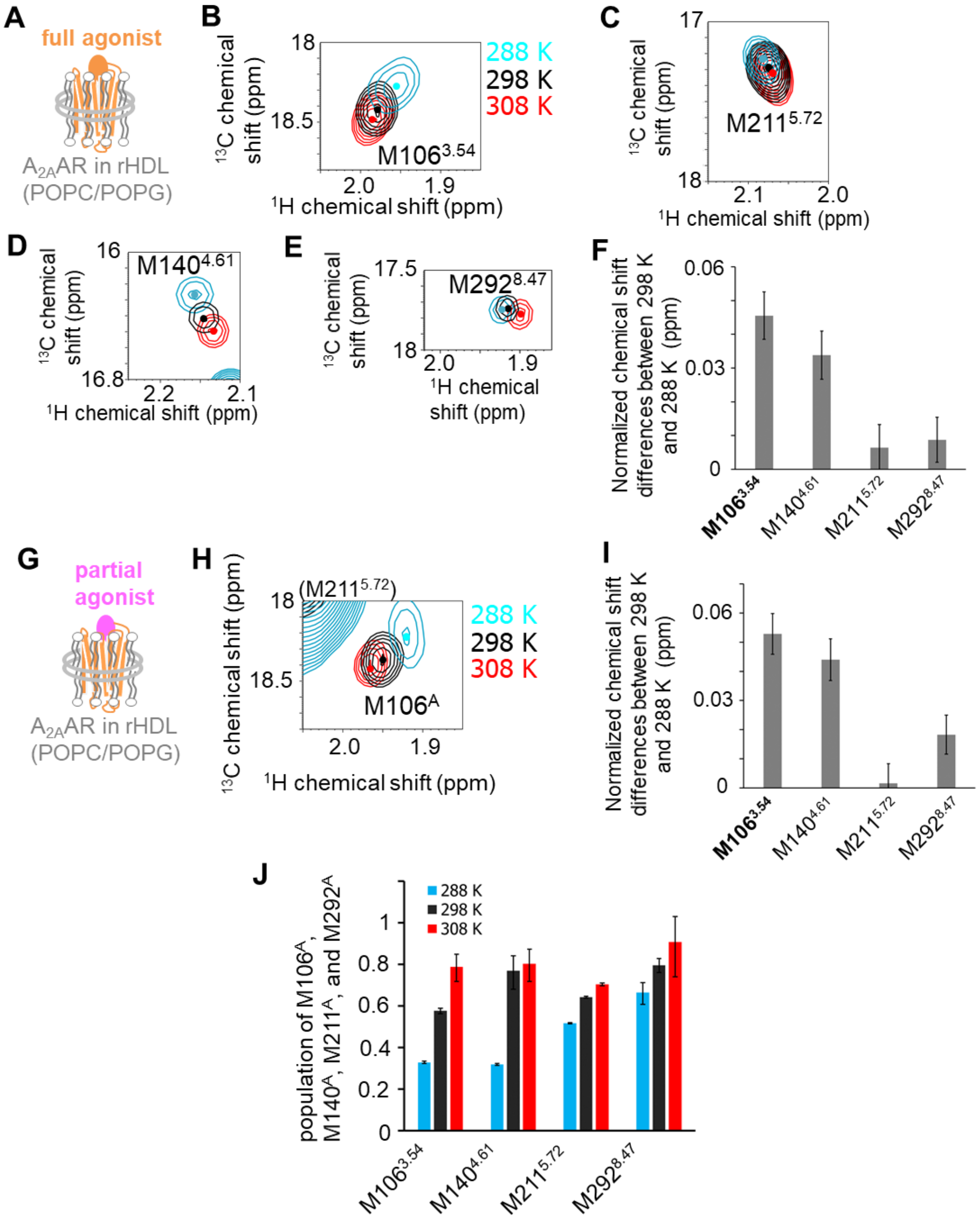

Fig. S5. NMR spectra of A_{2A}AR in rHDL(POPC/POPG) at various temperatures. (A) Schematic diagram of A_{2A}AR in rHDL(POPC/POPG) bound to the full agonist. **(B-E)** Overlaid ^{1}H-^{13}C HMQC spectra of the [[α,β,β-^{2}H, methyl-^{13}C] Met, u-^{2}H] A_{2A}AR/I106M/I292M in rHDL(POPC/POPG) bound to the full agonist, recorded at 298 K (black), 288 K (cyan), and 308 K (red). In (B), (C), (D), and (E), only the regions with the M106$^{3.54}$, M140$^{4.61}$, M292$^{8.47}$, and M211$^{5.72}$ methyl resonances are shown, respectively, and the centers of the methionine resonances are indicated with dots. **(F)** Normalized chemical shift differences of the methionine methyl resonances of A_{2A}AR in rHDL(POPC/POPG) in the presence of a full agonist at 298 K and those at 288 K. **(G)** Schematic diagram of A_{2A}AR in rHDL(POPC/POPG) bound to the partial agonist. **(H)** Overlaid ^{1}H-^{13}C HMQC spectra of the [[α,β,β-^{2}H, methyl-^{13}C] Met, u-^{2}H] A_{2A}AR/I106M/I292M in rHDL(POPC/POPG) bound to the partial agonist, recorded at 298 K (black), 288 K (cyan), and 308 K (red). Only the regions with M106$^{3.54}$ methyl resonances are shown. **(I)** Normalized chemical shift differences of the methionine

methyl resonances of $A_{2A}AR$ in rHDL(POPC/POPG) in the presence of a full agonist at 298 K and those at 288 K. **(J)** Intensity ratios of the $M106^A$, $M140^A$, $M211^A$, and $M292^A$ signals, relative to those of the $M106^I$, $M140^I$, $M211^I$, and $M292^I$ signals, at 288 K, 298 K, and 308 K. The error bars were calculated, based on the signal-to-noise ratios. In (F) and (I), the normalized chemical shifts and the errors were calculated in manners similar to Fig. 3F.

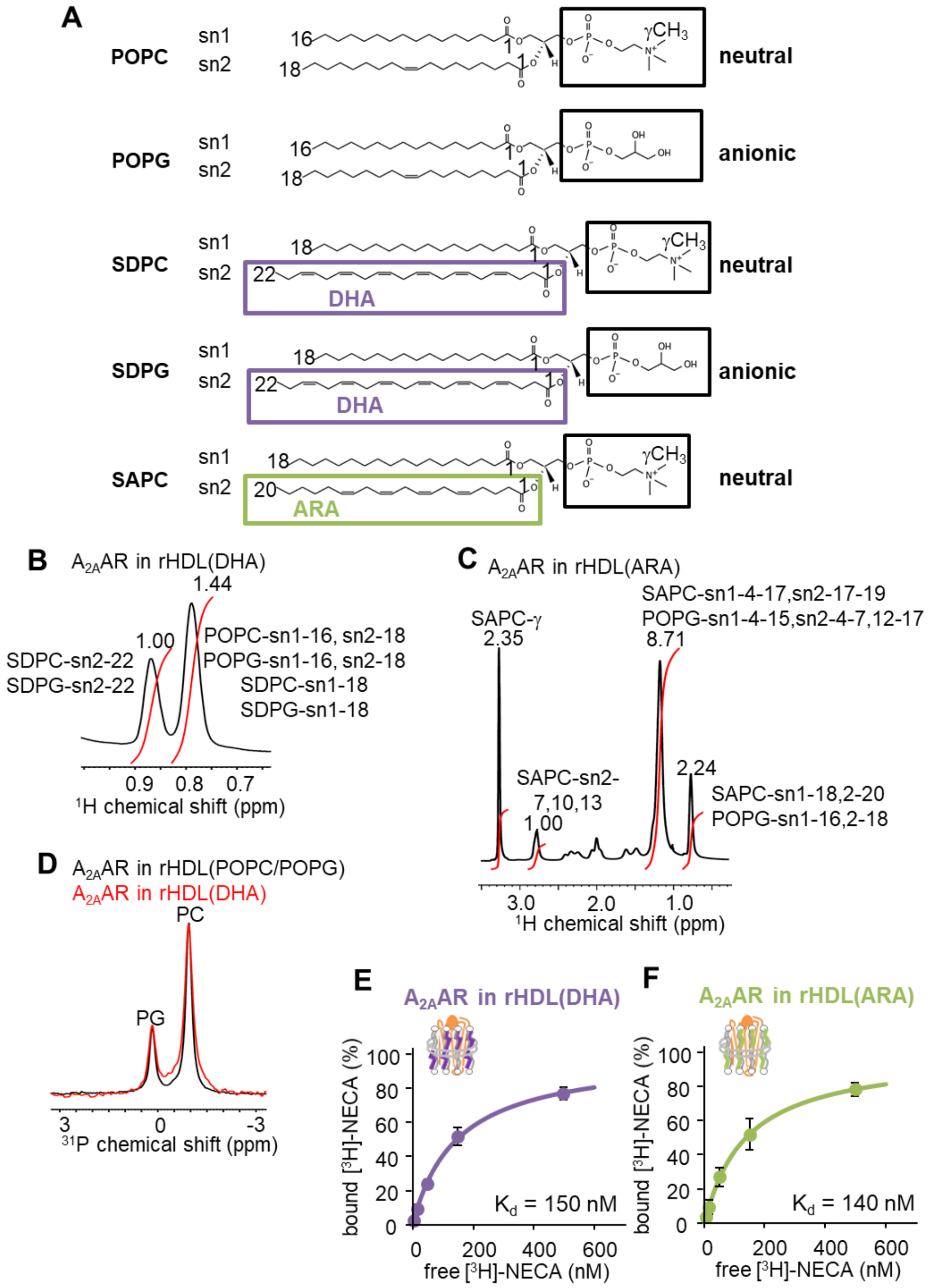

Fig. S6. Lipids used for the reconstitution of $A_{2A}AR$ into rHDLs. (A) Chemical structures of 1-palmitoyl-2-oleoyl-phosphatidylcholine (POPC), 1-palmitoyl-2-oleoyl-phosphatidylglycerol (POPG), 1-stearoyl-2-docosahexanoyl-phosphatidylcholine (SDPC), 1-stearoyl-2-docosahexanoyl-phosphatidylglycerol (SDPG), and 1-stearoyl-2-arachidonoyl-phosphatidylcholine (SAPC). **(B)** ^1H NMR spectrum of $A_{2A}AR$ in rHDL(DHA). The regions with methyl groups are shown. **(C)** ^1H NMR spectrum of $A_{2A}AR$ in rHDL(ARA). The regions with methyl and methylene groups are shown. **(D)**

Overlaid ^{31}P NMR spectra of $A_{2A}AR$ in rHDL(POPC/POPG) (black) and $A_{2A}AR$ in rHDL(DHA) (red). The assignments and the integration traces are indicated in (B)–(D) and (B)–(C), respectively. **(E, F)** Saturation curves for the [^{3}H]-full agonist (NECA) binding to $A_{2A}AR$ in rHDL(DHA) (E) and $A_{2A}AR$ in rHDL(ARA) (F). Each dataset is from triplicate experiments, and represents the average ± standard deviation.

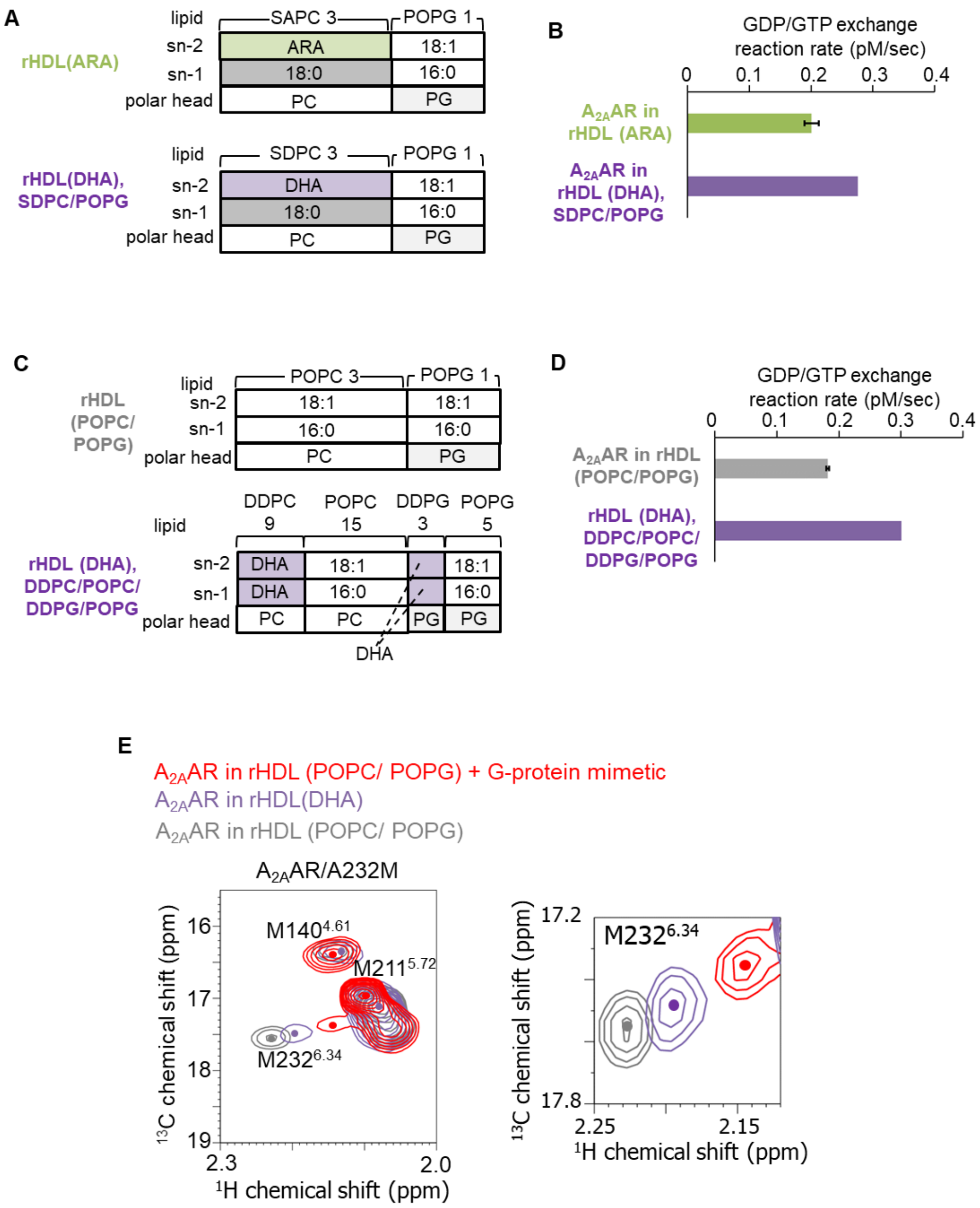

Fig. S7. Signaling activity and conformation of A_{2A}AR in rHDL. (A)-(B) Signaling activities of A_{2A}AR in rHDL(ARA) and A_{2A}AR in rHDL using lipids with SDPC and SDPG. **(C)-(D)** Signaling activities of A_{2A}AR in rHDL(POPC/POPG) and A_{2A}AR in rHDL using lipids with DDPC, POPC, DDPG, and POPG. (A) and (C) Schematic diagrams of the composition of the polar head and acyl chains at sn-1 and sn-2 positions of the lipids utilized for the preparation of A_{2A}AR in rHDLs. (B) and (D) [^{35}S]-GTPγS binding to complexes of the purified G protein and A_{2A}AR in rHDL. Data and errors of A_{2A}AR in rHDL(ARA) and A_{2A}AR in rHDL(POPC/POPG) in (B) and (D) are the same as those in Fig. 2B. **(E)** Overlaid ^{1}H-^{13}C HMQC spectra of A_{2A}AR/A232M, labeled with [[α,β,β-^{2}H, methyl-^{13}C] Met, u-^{2}H] and embedded in rHDL(POPC/POPG) (gray) and rHDL(DHA) (purple), and those of the ternary complex (red). Only the regions with M232$^{6.34}$ methyl resonances are shown in the right panel. The methionine methyl resonances are indicated, and the centers are indicated with dots.

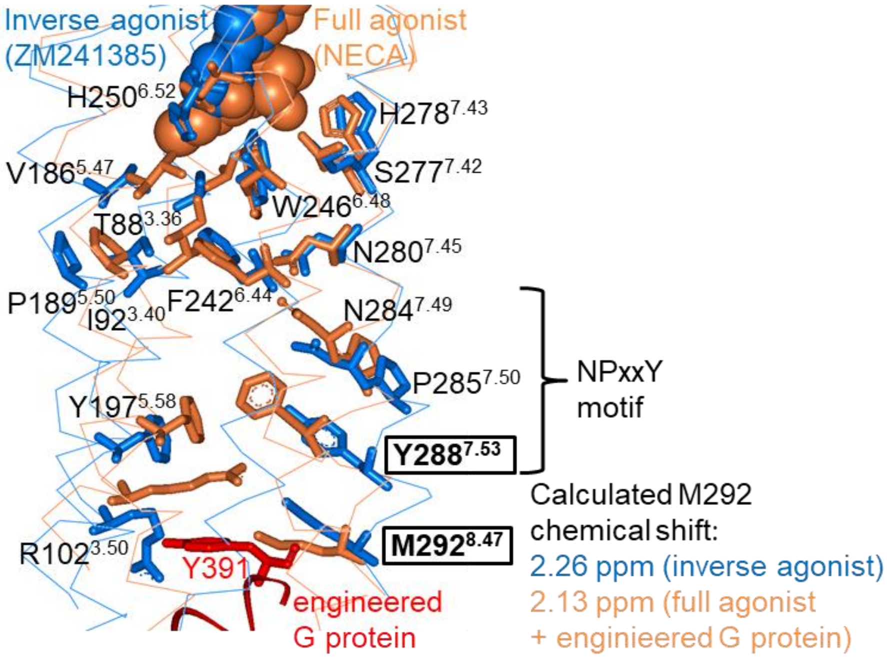

Fig. S8. Conformational changes in the TM region of A_{2A}AR upon activation. Shown are the crystal structures of A_{2A}AR complexes with the inverse agonist ZM241385 (PDB code: 3EML) (blue), and with the full agonist NECA and an engineered G protein (PDB code: 5G53), overlaid for the best fit of TM1 and TM2; the drawing represents a side view with the extracellular surface at the top. TM3, TM5, TM6, and TM7 are shown as blue or orange Cα traces, and the side chains of T88$^{3.36}$, I92$^{3.40}$, R102$^{3.50}$ V186$^{5.47}$, P189$^{5.50}$, Y197$^{5.58}$, F242$^{6.44}$, W246$^{6.48}$, H250$^{6.52}$, S277$^{7.42}$, H278$^{7.43}$, N280$^{7.45}$, N284$^{7.49}$ P285$^{7.50}$, Y288$^{7.53}$, and M292$^{7.54}$ are depicted by blue or orange sticks. Ligands are depicted by blue or orange CPK models. The engineered G protein is shown as a red ribbon, and Y391 is depicted by red sticks. The I292M mutation was introduced into the crystal structures with the MODELLER program. Ring current shifts, calculated from the coordinates of Y288$^{7.53}$ and M292$^{8.47}$ in the crystal structures, are indicated.

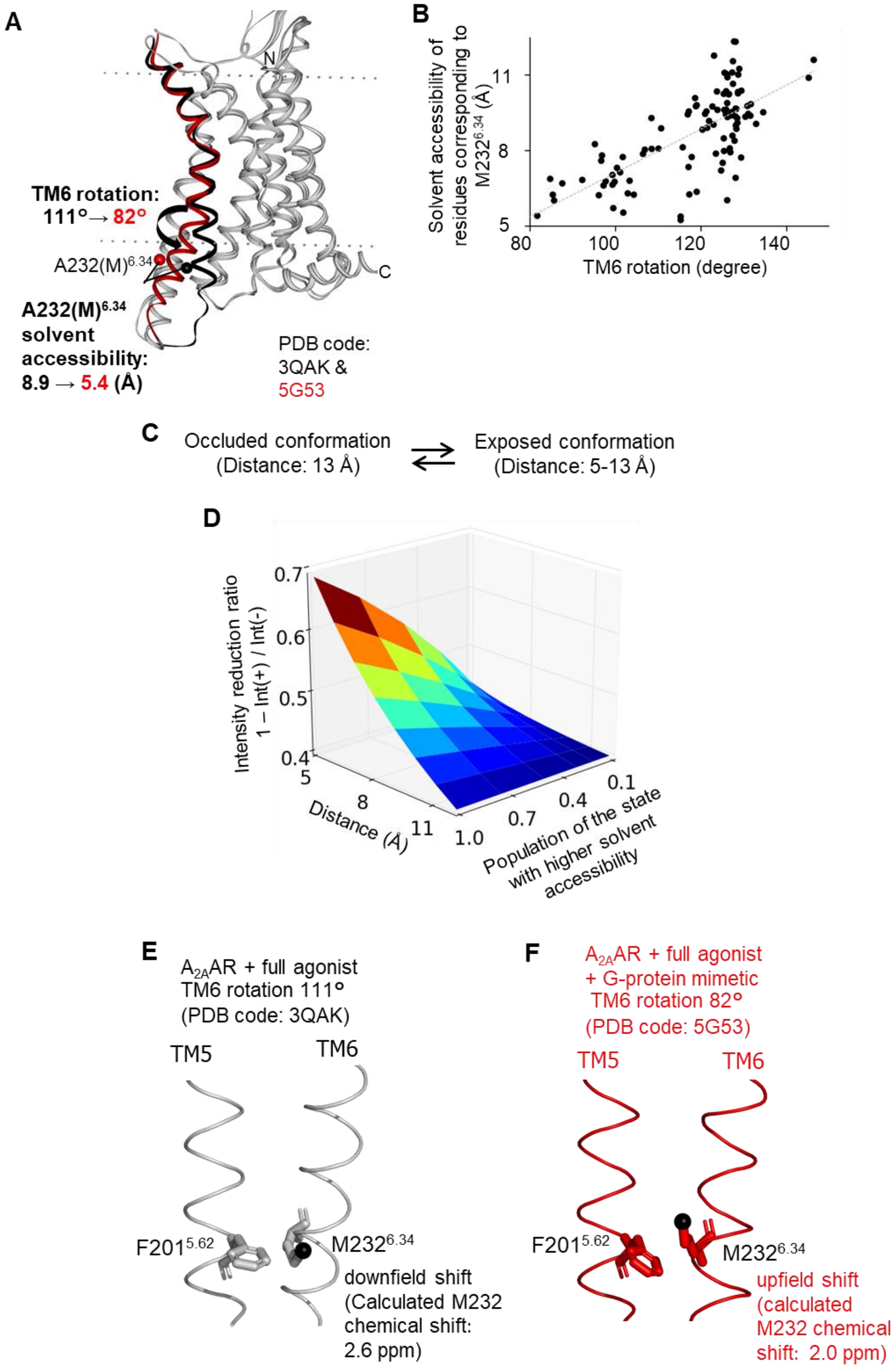

Fig. S9. Conformation of TM6. (A) Overlaid crystal structures of $A_{2A}AR$ exhibiting different TM6 rotation angles (PDB codes: 3QAK and 5G53). TM6 is black or red, and the Cβ atoms of $A232^{6.34}$, in which methionine was introduced by the A232M mutation in this study, are represented by black or red spheres. The $A232^{6.34}$ solvent accessibilities and the TM6 rotation angles are shown. The membrane positions generated by the orientations of the proteins in the membrane (OPM) database are indicated. **(B)** Plots of the TM6 rotation angles versus the solvent accessibilities of the residues corresponding to $A232^{6.34}$, represented as the closest approach distances between the Cβ of each residue and the hypothetical Gd-DTPA-DMA atoms, calculated from 118 GPCR crystal structures with hypothetical atoms corresponding to Gd-DTPA-DMA placed in the solvent. The

linear fit of the data is shown as a gray line. The TM6 angles are the angles between the vectors connecting the Cα and Cβ atoms of the residue corresponding to $A232^{6.34}$ of $A_{2A}AR$ and the vectors connecting the mean coordinates of the TM1, TM2, and TM4 atoms on the extracellular side and those on the intracellular side. **(C)** Schematic diagrams of the conformational equilibrium models utilized for the simulation. **(D)** Plots of the calculated signal intensity reduction ratios with various population of the state with higher solvent accessibility and the distances of the closest approaches between the observed atoms and the paramagnetic centers. **(E-F)** Crystal structures of $A_{2A}AR$ with a full agonist in the absence (E) and presence (F) of a G-protein mimetic. The A232M mutation was introduced into the crystal structures with the MODELLER program. TM5 and TM6 are shown as gray or red ribbons, and F201 and M232 are depicted by gray or red sticks. Cε atoms of M232 are depicted by gray or red spheres.

Table S1. [^{35}S]-GTPγS binding to complexes of purified G protein and $A_{2A}AR$ in rHDL(POPC/POPG) with 0, 0.5, 1, and 2 mol% trifluoroethanol (TFE), in the presence of the full agonist.

Concentration of TFE	Initial rate of the production of the GTPγS and G protein complex (pM/sec)
0%	0.21
0.5%	0.19
1%	0.20
2%	0.17
